# Lifestyle and genetic risk of chronic liver disease in metabolically healthy and unhealthy individuals from the general population

**DOI:** 10.1016/j.jhepr.2024.101105

**Published:** 2024-04-26

**Authors:** Isabel Drake, Alice Giontella, Mariam Miari, Kristina Önnerhag, Marju Orho-Melander

**Affiliations:** 1 Department of Clinical Sciences in Malmö, Lund University, Malmö, Sweden; 2 Skåne University Hospital, Malmö, Sweden; 3 Gastroenterology Research Unit, Department of Clinical Sciences in Malmö, Lund University, Malmö, Sweden; 4 Department of Surgery and Gastroenterology, Skåne University Hospital, Malmö, Sweden

**Keywords:** chronic liver disease, metabolic dysfunction-associated steatotic liver disease, cirrhosis, lifestyle, diet, polygenic risk

## Abstract

**Background & Aims:**

It is unclear to what extent lifestyle and genetic factors affect the incidence of chronic liver disease (CLD) in the general population and if lifestyle affects CLD independently of underlying cardiometabolic perturbations and genetic predisposition.

**Methods:**

We examined 27,991 men and women aged 44-73 years from the Malmö Diet and Cancer Study recruited between 1991-1996 and followed until the end of 2020 using registry linkage (median follow-up time 25.1 years; 382 incident first-time CLD events). Associations between cardiometabolic factors, polygenic risk scores (PRSs), and lifestyle factors in relation to CLD were examined using multivariable Cox proportional hazards regression models.

**Results:**

The incidence of CLD increased with number of cardiometabolic risk factors (the hazard ratio per each additional cardiometabolic risk factor was 1.33; 95% CI 1.21-1.45; *p =* 5.1 x 10^-10^). Two novel PRSs for metabolic dysfunction-associated steatotic liver disease and a PRS for cirrhosis were associated with higher risk of CLD but provided marginal predictive utility on top of other risk factors and compared to the *PNPLA3* rs738409 genetic variant. An unhealthy lifestyle (high alcohol intake, current smoking, physical inactivity and unhealthy diet) markedly increased the risk of CLD (hazard ratio 3.97, 95% CI 2.59-6.10). Observed associations between examined lifestyle factors and CLD were largely independent of cardiometabolic perturbations and polygenic risk.

**Conclusions:**

We confirmed the importance of cardiometabolic dysfunction in relation to risk of CLD in the general population. Lifestyle risk factors were shown to be independently associated with CLD and added predictive information on top of cardiometabolic risk factors. Information on the polygenic risk of liver disease does not currently improve the prediction of CLD in the general population.

**Impact and implications:**

This large population-based prospective study suggests largely independent roles of cardiometabolic, lifestyle, and genetic risk factors in the development of chronic liver disease. Findings strengthen the evidence base for a beneficial effect of modification of high-risk lifestyle behaviors in the primary prevention of chronic liver disease in the general population.

## Highlights

• Incidence of lifestyle-related chronic liver disease increases substantially with increasing number of cardiometabolic risk factors.
• Novel polygenic risk scores for MASLD and cirrhosis were associated with higher risk of chronic liver disease. However, they added little predictive information on top of cardiometabolic and lifestyle risk factors.
• The presence of modifiable lifestyle risk factors substantially increased the risk of chronic liver disease, regardless of underlying cardiometabolic perturbations or genetic predisposition.

## Graphical abstract

**Figure:**
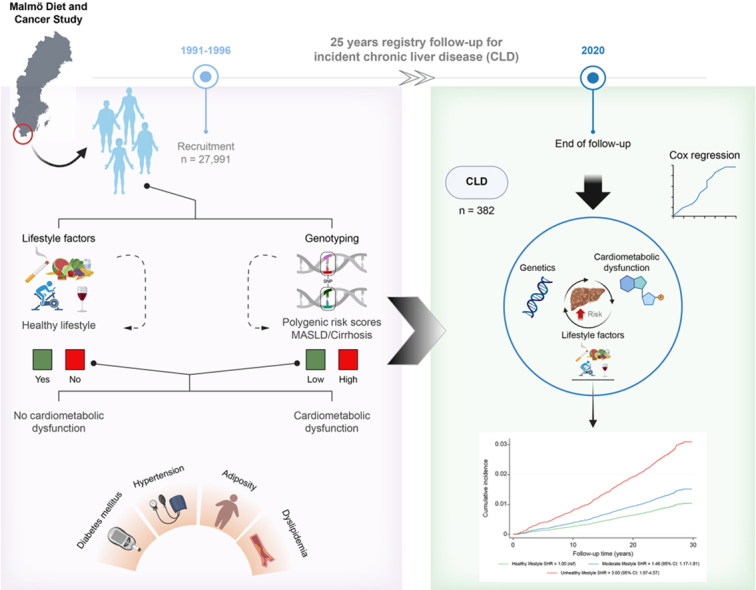

## Introduction

Liver diseases accounts for over two million deaths per year.^1^ The burden of liver diseases in Europe continues to grow, owing primarily to excessive alcohol consumption and the increasing prevalence of obesity.^2^ Both obesity and excessive alcohol consumption are important causes of steatotic liver disease (SLD). The worldwide prevalence of metabolic dysfunction-associated SLD (MASLD) was recently estimated to be over 30%, with a geographical distribution that varies depending on factors such as ethnicity, genetic predisposition and lifestyle factors.^3^^,^^4^ For alcohol-associated liver disease (ALD), a recent systematic review suggested a prevalence of 3.5% in the general population. However, in groups with alcohol use disorder the prevalence of ALD is approximately 51%.^5^ In the new revision of the SLD nomenclature, MASLD with alcohol intake (MetALD) was defined as a specific subgroup of SLD.^3^ This highlights the important notion that alcohol intake and metabolic dysfunction are rarely mutually exclusive risk factors in the clinical setting.^6^

Increased fine-tuning of the classification of heterogeneous diseases such as SLD based on disease characteristics and etiology can greatly aid the targeted treatment of established disease. However, for primary prevention such sub-classification tends to be less useful since the population will always be at risk of different outcomes and the presence of multiple exposures may need joint consideration. Patients with SLD are at risk of developing chronic liver disease (CLD) including cirrhosis and hepatocellular carcinoma (HCC). Due to the poor prognosis of CLD it is important to identify useful risk markers to adequately identify risk groups that may benefit from intervention or screening.

MASLD has until recently been considered oligogenic which is in notable contrast to other cardiometabolic diseases and traits. Previous genome-wide association studies (GWAS) have typically identified only a small subset of genetic risk variants for MASLD. However, recent efforts with increased GWAS sample sizes have expanded our understanding of the potentially polygenic nature of MASLD^7^^,^^8^ and cirrhosis.^9^ Several lifestyle factors have also been proposed to play a role in development and management of MASLD as well as CLD,[10], [11], [12] however, few prospective studies have examined the impact of lifestyle factors on CLD in the general population.^13^ It has further been reported that individuals with genetic predisposition to liver disease are at higher risk of liver damage due to exogenous risk factors.^14^

In this study, we wanted to examine how lifestyle factors associate with CLD and specifically examine if these associations differ based on underlying cardiometabolic perturbations or genetic predisposition. We further wanted to assess the potential utility of recently identified polygenic risk scores (PRSs) for the prediction of CLD in the general population.

## Materials and methods

### Study population

**Fig. 1:**
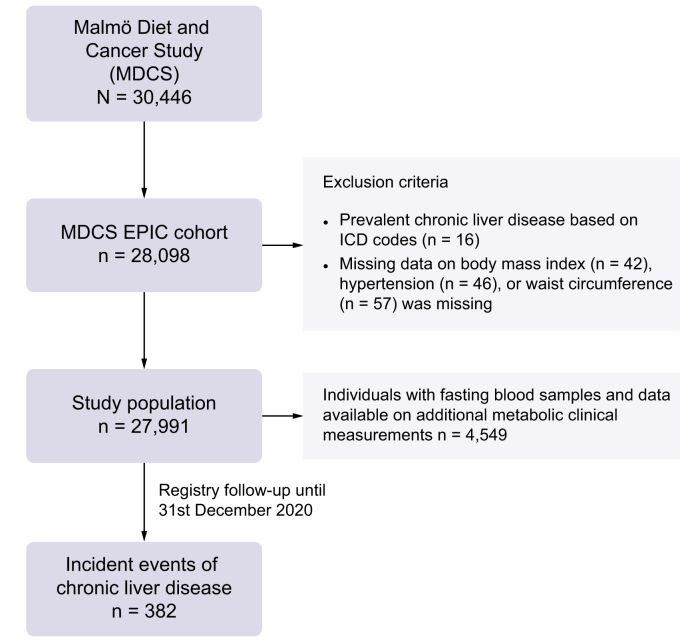
Flow chart of the study population. MDCS, Malmö Diet and Cancer Study.

The Malmö Diet and Cancer Study (MDCS) is a population-based prospective cohort study.^15^^,^^16^ In short, during 1991-1996, all inhabitants of the city of Malmö (Southern Sweden) aged between 44-73 years were invited to join the study. In total, the MDCS recruited 30,446 men and women (participation rate approximately 40%).^17^ Among the MDCS participants, 28,098 participants completed the majority of baseline examinations including a detailed dietary assessment and constitute a part of the EPIC (European Prospective Investigation into Nutrition and Cancer) cohort. Baseline examinations included direct measurements and donation of non-fasting blood samples stored in a biobank, an extensive baseline questionnaire covering lifestyle, socioeconomic and disease history, and dietary assessment using a modified diet history method.^16^ Between 1991 and 1994, every other participant in the MDCS was asked to join a sub-cohort (n = 6,103) in which participants underwent additional examinations including donation of fasting blood samples.^18^ A flow chart of the study population including exclusion criteria for the current study is shown in Fig. 1.

### Ascertainment CLD at baseline and during follow-up

A composite endpoint of CLD based on ICD-9/10 codes in the National Patient Register and the Swedish Cause of Death Register was used as the primary endpoint (Table S1). Participants were followed from baseline through register linkage using their Swedish personal identification number until first incident event of CLD, emigration (<0.5%), death, or 31^st^ December 2020. Diagnoses classified as CLD included acute and subacute liver failure (ICD-9:570 or ICD-10 K72.0), chronic liver failure (ICD-9 572.8 or ICD-10 K72.1), liver failure (ICD-10 K72.9, K70.4), cirrhosis (ICD-9 571.5 or ICD-10 K74.6, K70.3), portal hypertension (ICD-9 571.5 or ICD-10 K76.6), hepatorenal syndrome (ICD-9 572.4 or ICD-10 K76.7), esophageal varices (ICD-9 456 or ICD-10 I85.0, I85.9), ascites (ICD-9 789.5 or ICD-10 R18.9), liver encephalopathy (ICD-9 572.2), hepatocellular carcinoma (ICD-9 155 or ICD-10 C22.0), and liver transplantation (ICD-10 JJC00, JJC10, JJC20, DJ005, DJ006, JJC30, JJC40). For diagnosis of ascites, only cases with a subsequent diagnosis of another CLD event were included (n = 32), while the remainder were censored at the time of ascites diagnosis (n = 322). No participants had a prevalent diagnosis of chronic viral hepatitis and/or other non-lifestyle-related causes of liver disease at baseline (ICD-10 codes [or corresponding codes using earlier ICD-version] B18, B19, E83.0, E83.1, K71, K74.3, K74.4, K74.5, K75.2, K75.3, K75.4, K75.8, K75.9). Individuals with any incident diagnosis of chronic viral hepatitis and/or other non-lifestyle-related cause of liver disease were censored at the time of diagnosis (n = 307 of whom 82 were later diagnosed with CLD) and were not included in analysis of the primary endpoint. Sensitivity analyses were also performed to examine the association with specific liver-related outcomes including cirrhosis and HCC, as well as SLD (ICD-10 K76.0), which was not included in the primary endpoint.

### Ascertainment of cardiometabolic health at baseline examinations

A detailed description of baseline assessments of anthropometric, cardiometabolic and blood measurements is provided in the Supplementary Material. Study participants were grouped according to their cardiometabolic health at baseline based on the number of fulfilled adult criteria for metabolic dysfunction as outlined in the definition of MASLD.^3^^,^^19^ An unhealthy cardiometabolic status was defined as fulfilling at least one out of five criteria: 1) BMI ≥25 kg/m^2^, 2) waist circumference >94 cm for men or >80 cm for women, 3) prevalent diabetes mellitus, 4) presence of hypertension (blood pressure ≥130/85 mmHg) and/or use of anti-hypertensive medication, or 5) use of lipid-lowering medications. Among individuals with fasting blood samples taken at baseline, analyses were performed to examine additional cardiometabolic risk factors including plasma triglycerides (mmol/l), high-density lipoprotein-cholesterol (mmol/L), fasting glucose (mmol/L), homeostatic model assessment for insulin resistance (HOMA-IR), and plasma high-sensitivity C-reactive protein level (mg/L).

### Lifestyle variables

A detailed description of the included lifestyle variables is found in the Supplementary Material. We examined four modifiable lifestyle factors: alcohol consumption, smoking status, physical activity and diet. Smoking status was categorized as never, former or current (including irregular). Alcohol consumption was categorized as zero, low, moderate or high. Leisure-time physical activity level was categorized by dividing participants into sex-specific quartiles of a physical activity score based on self-reported time spent on leisure-time physical activities. We examined three proposed ‘healthy’ dietary components (dietary fiber, fruits and vegetables, and coffee) and two ‘unhealthy’ components (sugar-sweetened beverages and red and processed meat). A diet risk score was constructed to reflect overall diet by dividing participants into tertiles of intakes of the five components. The score was constructed by assigning points (1, 2, or 3) based on tertiles of energy-adjusted intakes and adding the five components together into a total score (ranging from 5-15 points). Low intakes of dietary fiber, fruit and vegetables and coffee were given low points whereas low intakes of sugar-sweetened beverages and red and processed meats were given high points based on previously reported directions of effect (see Supplementary Material for more information). The total score aimed to reflect a range from unhealthy (low points) to healthy (high points) diet. To assess the combined impact of overall lifestyle we constructed a lifestyle risk score where one point each were received for current smoking, high alcohol consumption, low physical activity (quartile 1), and unhealthy diet (diet risk score 5-7 points), where 0 points reflected absence and 4 points reflected presence of all four risk factors. Participants with no lifestyle risk factors were classified as healthy whereas those with 3-4 risk factors were classified as unhealthy.

### Genotyping and PRSs

Genotyping of MDCS participants was performed using the Illumina GSA v1 genotyping array and details of the genotyping and quality control procedures have been described in detail previously.^20^ We considered genetic variants previously associated with MASLD and cirrhosis for construction of three PRSs. The PRS-MASLD included 16 independent genetic variants associated with MASLD in the European ancestry-only meta-analysis by Chen *et al.*.^8^ The PRS-cALT included 17 genetic variants identified using unexplained chronically elevated ALT (cALT) levels as a proxy for MASLD that showed concordant effect estimates in cohorts with imaging or histology verified MASLD in a study by Vojkovic *et al.*.^7^ The PRS-cirrhosis included 12 genetic variants associated with cirrhosis in a study by Emdin *et al.*.^9^ Genetic variants in four genes (*PNPLA3, TM6SF2, MARC1* and *APOE*) were included in all three scores. Genetic variants in two additional genes (*TOR1B* and *SERPINA1*) were included in two of the scores whereas the remaining variants were specific to the respective PRSs. Genotypes of two variants, MTTP rs138765179 in the PRS-MASLD score and HMBS rs1799992 in the PRS-cirrhosis score, were not available and we therefore used two proxy variants (r^2=^1 and D’=1). A list of all included variants as well as weights used to construct the PRSs are shown in Table S2. All genetic variants were coded as 0, 1 or 2 for non-carriers, heterozygous carriers, and homozygous carriers of the minor allele, respectively. The PRSs were calculated by summing the number of minor alleles and weighting by their corresponding effect sizes (reported z-scores, natural log odds ratios or beta coefficients). The effect of the individual genetic variants on CLD by genotype and the per minor allele effects were also examined.

### Statistical analysis

Baseline characteristics of the study population and differences by cardiometabolic health status at baseline were assessed using the t-test or the Kruskal-Wallis test for continuous variables and the chi-square test for categorical variables. The cumulative incidence of CLD by number of fulfilled cardiometabolic criteria was estimated using a competing risk regression model based on the Fine and Gray method taking into account the competing risk of non-CLD deaths, and with adjustment for age and sex. A Cox proportional hazards regression model with follow-up time as the underlying time-metric was used to assess hazard ratios (HRs) and 95% CIs for CLD by differences in baseline cardiometabolic, lifestyle and genetic risk factors. An age- and sex-adjusted Cox proportional hazards model was used as a basic model to assess the role of each risk factor individually, and a mutually adjusted model including all lifestyle risk factors simultaneously was constructed to identify lifestyle risk factors independently associated with CLD. The cumulative incidence of CLD by the lifestyle risk score (healthy, moderate, unhealthy) was estimated using a competing risk regression model with adjustment for age, sex, and educational level. To examine the association between PRS-MASLD, PRS-cirrhosis and PRS-cALT with CLD, we fitted restricted cubic splines to Cox regression models adjusting for age and sex and assessed the HRs and 95% CIs per standard deviation (SD) increase. Heterogeneity in the associations between individual lifestyle risk factors in relation to CLD by number of cardiometabolic risk factors at baseline was examined by including the multiplicative interaction terms in the age- and sex-adjusted models and in the fully adjusted models. We further assessed multiplicative interactions between cardiometabolic and lifestyle risk factors with the *PNPLA3* rs738409 genetic variant, PRS-MASLD, PRS-cirrhosis and PRS-cALT in Cox regression models adjusting for age, sex, and educational level. To examine the predictive utility of cardiometabolic, lifestyle and genetic risk factors for CLD we calculated the Harrell’s C-statistic and used the likelihood ratio test to assess significant model improvement. All multivariable analyses were complete case analyses and thus total number differed slightly between analyses due to missing data on some covariates, with sample sizes ranging from 27,991 to 26,725 in analyses that included all covariates. Covariates with missing data included educational level (n = 70), smoking status (n = 12), physical activity (n = 186) and genotype data (n = 1025). As a sensitivity analysis, we examined associations with specific liver-related outcomes including cirrhosis, HCC and SLD. Deviation from the proportional hazards assumption in all Cox regression models was tested using the Schoenfeld test; no significant deviations were noted (all *p* >0.10). Non-linear effects by non-categorized continuous variables were tested by fitting restricted cubic splines and the likelihood ratio test was used to test for significant deviations from linearity; no significant deviations were noted (all *p* >0.10). Continuous variables with a skewed distribution were transformed using a natural log transformation and standardized to a normal distribution with mean 0 and SD of 1 and the effects per 1 SD increase were estimated. All tests were two-sided and *p* values <0.05 were considered statistically significant. All analyses were performed in Stata/SE Version 15.1 and R version 4.3.1 (The R Foundation for Statistical Computing Platform).

## Results

### Description of study population

During a median follow-up time of 25.1 years (IQR 18.6-27.0 years), 382 incident events of CLD occurred. Baseline characteristics of participants are shown in Table 1. In the study population, 4.5% had diabetes mellitus at baseline and mean BMI was 25.7 kg/m^2^ (SD = 4.0). At baseline, 55% were defined as hypertensive and 3.2% reported current use of lipid-lowering drugs (Table 1). In total 75.7% of the study population fulfilled at least one criteria of metabolic dysfunction (27.9% fulfilled one criterium only, 21.8% fulfilled two criteria, and 26.0% fulfilled three or more criteria).

**Table 1:** Baseline lifestyle, genetic and metabolic characteristics of the MDCS (N = 27,991) and the MDCS-CC (N = 4,549) overall and by categorization of participants based on metabolic health status at baseline.

Characteristic	All participants	Metabolically healthy	Metabolically unhealthy	*p* value∗
Number of participants, n (%)	27,991	6,817 (24.4)	21,174 (75.7)	—
Number of incident CLD cases, n (%)	382 (1.4)	58 (0.9)	324 (1.5)	<0.0001
Age, years (SD)	58.1 (7.6)	55.3 (7.2)	59.0 (7.5)	<0.0001
Male sex, n (%)	11,020 (39.4)	1,772 (26.0)	9,248 (43.7)	<0.0001
Prevalent diabetes mellitus, n (%)	1,245 (4.5)	0 (0)	1,245 (5.9)	<0.0001
Body mass index, kg/m^2^	25.7 (4.0)	22.3 (1.8)	26.9 (3.8)	<0.0001
Waist circumference, cm (SD)	84.1 (12.9)	73.8 (7.9)	87.4 (12.5)	<0.0001
Hypertension, n (%)	15,383 (55.0)	0 (0)	15,383 (72.7)	<0.0001
Use of lipid-lowering drugs, n (%)	903 (3.2)	0 (0)	903 (4.3)	<0.0001
Educational level, n (%)				<0.0001
Elementary school	11,731 (42.0)	2,116 (31.1)	9,615 (45.5)	
Middle school	7,299 (26.1)	1,916 (28.2)	5,383 (25.5)	
High school	4,924 (17.6)	1,374 (20.2)	3,550 (16.8)	
University degree	3,967 (14.2)	1,399 (20.6)	2,568 (12.2)	
Smoking status, n (%)				<0.0001
Never	10,612 (37.9)	2,467 (36.2)	8,145 (38.5)	
Former	9,461 (33.8)	1,986 (29.1)	7,475 (35.3)	
Current	7,906 (28.3)	2,363 (34.7)	5,543 (26.2)	
Alcohol consumption, n (%)				<0.0001
Zero	1,699 (6.1)	333 (4.9)	1,366 (6.5)	
Low	20,308 (72.6)	5,026 (73.7)	15,282 (72.2)	
Moderate	4,780 (17.1)	1,216 (17.8)	3,564 (16.8)	
High	1,204 (4.3)	242 (3.6)	962 (4.5)	
Low physical activity score, n (%)	6,952 (25.0)	1,480 (21.8)	5,472 (26.0)	<0.0001
Dietary fiber, g/1,000 kcal (IQR)	8.8 (7.2-10.7)	8.8 (7.3-10.7)	8.8 (7.2-10.7)	0.30
Fruit and vegetables, g/1,000 kcal (IQR)	161 (110-226)	162 (111-229)	161 (110-226)	0.22
SSB, g/1,000 kcal (IQR)	2.7 (0-42)	1.9 (0.0-37.0)	3.3 (0.0-44.1)	0.0007
Coffee, g/1,000 kcal (IQR)	204 (117-323)	211 (121-340)	202 (115-318)	<0.0001
Red/processed meat, g/1,000 kcal (IQR)	50.9 (27.6-66.1)	47.6 (33.9-62.1)	52.1 (38.7-67.3)	<0.0001
*PNPLA3* rs738409 GG-genotype, n (%)	1,319 (4.5)	288 (4.4)	916 (4.5)	0.81
Top decile of PRS-MASLD, n (%)	2,696 (10)	627 (9.5)	2,069 (10.1)	0.72
Top decile of PRS-cirrhosis, n (%)	2,692 (10)	594 (9.0)	2,098 (10.3)	0.012
Top decile of PRS-cALT, n (%)	2,693 (10)	627 (9.5)	2,066 (10.1)	0.16
Number of participants, n (%)	4,549	1,157 (25.4)	3,392 (74.6)	—
Fasting plasma glucose, mmol/L (IQR)	5.4 (5.1-5.8)	5.2 (5.0-5.6)	5.4 (5.1-5.8)	<0.0001
HbA1c, % (IQR)	4.8 (4.5-5.0)	4.7 (4.5-5.0)	4.8 (4.5-5.1)	0.0005
HOMA-IR (IQR)	1.3 (0.9-1.9)	1.0 (0.6-1.4)	1.5 (1.0-2.1)	<0.0001
LDL, mmol/L (IQR)	4.1 (3.5-4.8)	3.9 (3.3-4.6)	4.2 (3.5-4.8)	<0.0001
HDL, mmol/L (IQR)	1.3 (1.1-1.6)	1.5 (1.2-1.7)	1.3 (1.1-1.6)	<0.0001
Triglycerides, mmol/L (IQR)	1.1 (0.9-1.5)	1.0 (0.8-1.3)	1.2 (0.9-1.6)	<0.0001
hsCRP, mg/L (IQR)	1.3 (0.6-2.6)	0.9 (0.5-1.8)	1.5 (0.7-2.9)	<0.0001

cALT, chronically elevated ALT; CLD, chronic liver disease; HOMA-IR, homeostatic model assessment for insulin resistance; MASLD, metabolic dysfunction-associated steatotic liver disease; MDCS, Malmö Diet and Cancer Study; MDCS-CC -Malmö Diet and Cancer Study, Cardiovascular Cohort; PRS, polygenic risk score; SSB, sugar-sweetened beverage.

∗ *p* values for differences by metabolic health status from chi-square test for categorical variables (expressed as n (%)) and t-test for normally distributed continuous variables (expressed as mean (SD)) or Kruskal-Wallis test for continuous variables with skewed distribution (expressed as median (IQR)).

### Cardiometabolic risk factors

**Fig. 2:**
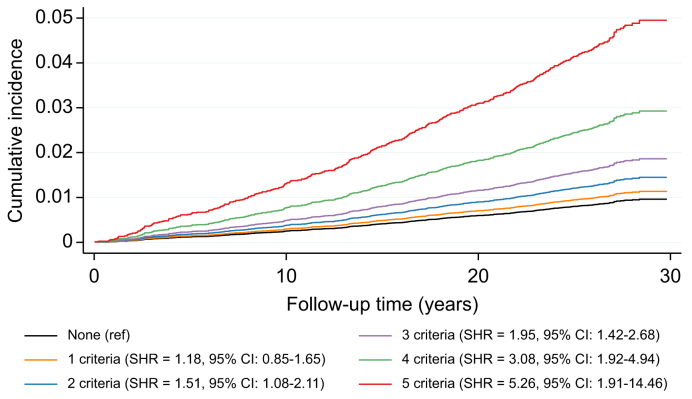
Cumulative incidence of CLD by a number of adult cardiometabolic criteria for MASLD in the MDCS. The cumulative incidence and the SHR with 95% CIs were estimated using a competing risk regression model accounting for non-CLD deaths as competing events and adjusting for age and sex. CLD, chronic liver disease; MASLD, metabolic dysfunction-associated steatotic liver disease; MDCS, Malmö Diet and Cancer Study; SHR, subdistribution hazard ratio.

The cumulative incidence of CLD increased by number of fulfilled cardiometabolic criteria (Fig. 2). After adjustment for age, sex, and educational level, the risk increase per number of fulfilled criteria was 1.33 (95% CI 1.21-1.45; *p* = 5.1 x 10^-10^) and individuals fulfilling all five cardiometabolic criteria had an 8-fold higher risk of CLD compared to those with none (HR 8.33; 95% CI 3.02-23.03; *p* = 4.4 x 10^-5^). The effect of individual cardiometabolic risk factors on risk of CLD was examined after adjustment for age- and sex as well as after adjustment for prevalent diabetes mellitus, BMI, hypertension, and use of lipid-lowering drugs (Table S3). In the fully adjusted model, prevalent diabetes mellitus (HR 2.22; 95% CI 1.56-3.15; *p* = 8.3 x 10^-6^), BMI (HR per SD increase = 1.26; 95% CI 1.13-1.40; *p* = 2.6 x 10^-5^), waist circumference (HR per SD increase = 1.93; 95% CI 1.49-2.50; *p* = 6.3 x 10^-7^) and HOMA-IR (HR per SD increase = 2.11; 95% CI 1.62-2.75; *p* = 2.8 x 10^-8^) were associated with increased risk of CLD.

### Lifestyle risk factors

**Fig. 3:**
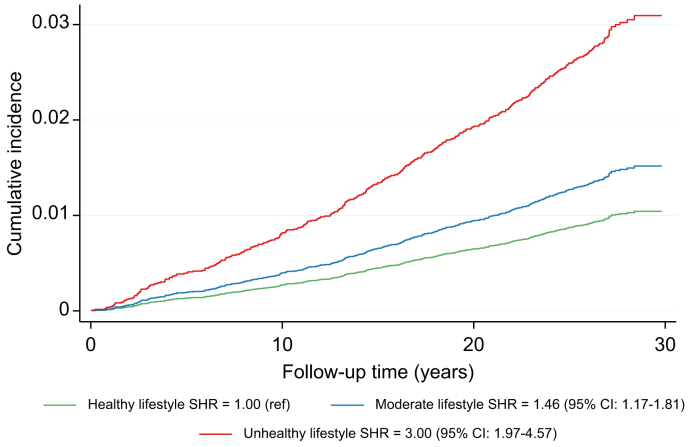
Lifestyle risk score in relation to incident CLD in the MDCS (N = 27,737). The plot displays the cumulative incidence function by categories of lifestyle risk score based on presence of high alcohol intake, current smoking, physical inactivity and unhealthy diet (healthy lifestyle=zero risk factors; moderate lifestyle=1-2 risk factors; unhealthy lifestyle=3-4 risk factors). SHR and 95% CIs were estimated using a competing risk regression model accounting for non-CLD deaths as competing events and adjusting for age, sex and educational level. CLD, chronic liver disease; MDCS, Malmö Diet and Cancer Study; SHR, subdistribution hazard ratio.

The cumulative incidence of CLD was higher among those with an unhealthy compared to healthy lifestyle (Fig. 3). After adjustment for age, sex, and educational level, an unhealthy compared to a healthy lifestyle was associated with increased risk of CLD (HR 3.97; 95% CI 2.59-6.10; *p* for trend across categories = 4.5 x 10^-10^). Additional adjustment for prevalent diabetes mellitus, BMI, waist circumference, hypertension and use of lipid-lowering drugs had a very modest attenuating effect on the observed risk estimate (HR 3.72; 95% CI 2.41-5.72; *p* for trend across categories = 7.2 x 10^-9^; data not tabulated). The risk estimates associated with an unhealthy lifestyle were stronger for SLD (HR 5.65; 95% CI 2.41-13.2; *p* = 6.7x10^-5^) and cirrhosis (HR 4.75; 95% CI 2.61-8.67; *p =* 1.0 x 10^-7^), but not HCC (HR 3.13; 95% CI 1.29-7.56; *p =* 0.011) (data not tabulated). All examined lifestyle risk factors were associated with a higher risk of CLD after mutual adjustment including current smoking (HR 1.71; 95% CI 1.33-2.19), high alcohol intake (HR 2.30; 95% CI 1.61-3.28), physical inactivity (HR 1.41; 95% CI 1.06-1.88), and an unhealthy diet (HR 1.53; 95% CI 1.02-2.30) (Table 2). Among the dietary components included in the diet risk score, we observed a protective effect of high fiber intake (HR per SD increase = 0.80; 95% CI 0.68-0.93) and coffee intake (HR per SD increase = 0.90; 95% CI 0.81-0.99). There was a non-significant tendency for a higher risk of CLD with higher intake of red and processed meats (HR 1.12; 95% CI 0.99-1.26; *p =* 0.061) (Table 2).

**Table 2:** Lifestyle risk factors for CLD in the MDCS (N = 27,991) and heterogeneity by number of cardiometabolic criteria at baseline.

Risk factor	n (cases)	HR (95% CI) Model 1∗	*p* value	HR (95% CI) Model 2∗∗	*p* value	*p*_interaction_†
Educational level						0.90 (0.62)
Elementary or lower	11,731 (190)	1.00 (ref)	ref	1.00 (ref)	ref	
Middle school	7,299 (87)	0.79 (0.61-1.03)	0.079	0.82 (0.63-1.06)	0.13	
High school	4,924 (75)	0.87 (0.66-1.13)	0.29	0.90 (0.69-1.19)	0.47	
University degree	3,967 (28)	0.43 (0.29-0.64)	3.9 x 10^-5^	0.47 (0.31-0.71)	2.7 x 10^-4^	
Smoking status						0.014 (0.58)
Never	10,612 (120)	1.00 (ref)	ref	1.00 (ref)		
Former	9,461 (115)	0.96 (0.74-1.25)	0.77	0.92 (0.70-1.19)	0.52	
Current	7,906 (146)	1.90 (1.49-2.43)	3.2 x 10^-7^	1.71 (1.33-2.19)	3.1 x 10^-5^	
Alcohol consumption						0.97 (0.80)
Zero	1,699 (27)	1.59 (1.06-2.36)	0.024	1.41 (0.93-2.14)	0.10	
Low	20,308 (243)	1.00 (ref)	ref	1.00 (ref)	ref	
Moderate	4,780 (73)	1.14 (0.87-1.49)	0.33	1.15 (0.88-1.50)	0.32	
High	1,204 (39)	2.43 (1.72-3.43)	4.7 x 10^-7^	2.30 (1.61-3.28)	4.3 x 10^-6^	
Physical activity score						0.50 (0.35)
Quartile 4	6,947 (83)	1.00 (ref)	ref	1.00 (ref)	ref	
Quartile 3	6,937 (99)	1.18 (0.88-1.58)	0.27	1.19 (0.88-1.59)	0.25	
Quartile 2	6,969 (75)	0.90 (0.66-1.24)	0.53	0.89 (0.65-1.22)	0.46	
Quartile 1	6,952 (121)	1.55 (1.17-2.05)	2.3 x 10^-3^	1.41 (1.06-1.88)	0.017	
Diet risk score						0.53 (0.36)
Healthy (13-15 points)	4,548 (39)	1.00 (ref)	ref	1.00 (ref)	ref	
Moderate (8-12 points)	19,778 (263)	1.34 (0.95-1.89)	0.092	1.18 (0.83-1.68)	0.34	
Unhealthy (5-7 points)	3,665 (80)	1.96 (1.31-2.91)	9.3 x 10^-4^	1.53 (1.02-2.30)	0.041	
Dietary fiber, per SD increase	27,991 (382)	0.73 (0.66-0.81)	1.2 x 10^-9^	0.80 (0.68-0.93)	3.7 x 10^-3^	0.11 (0.16)
Fruit/vegetables, per SD increase	27,991 (382)	0.82 (0.74-0.90)	6.9 x 10^-5^	1.06 (0.91-1.24)	0.43	0.88 (0.72)
SSB, per SD increase	27,991 (382)	1.01 (0.91-1.12)	0.84	0.97 (0.88-1.08)	0.58	0.42 (0.58)
Coffee, per SD increase	27,991 (382)	0.94 (0.86-1.04)	0.24	0.90 (0.81-0.99)	0.029	0.024 (0.040)
Red/processed meat, per SD increase	27,991 (382)	1.19 (1.06-1.33)	3.6 x 10^-3^	1.12 (0.99-1.26)	0.061	0.96 (0.38)

CLD, chronic liver disease; HR, hazard ratio; MDCS, Malmö Diet and Cancer Study; SD, standard deviation; SSB, sugar-sweetened beverage.

HRs and 95% CIs were estimated using Cox proportional hazards models.

∗ Model 1 adjusted for age and sex.

∗∗ Model 2 adjusted for age and sex further included all covariates in the table except for individual dietary components to assess mutually independent effects of lifestyle risk factors. For individual dietary components, model 2 was adjusted for all covariates in the table except diet risk score.

† *p* value for multiplicative interaction between individual risk factors in the table with number of fulfilled cardiometabolic criteria at baseline adjusting for age and sex, and *p* value for interaction with mutual adjustment for all included covariates as outlined for Model 2 in parenthesis.

### Genetic risk factors

**Fig. 4:**
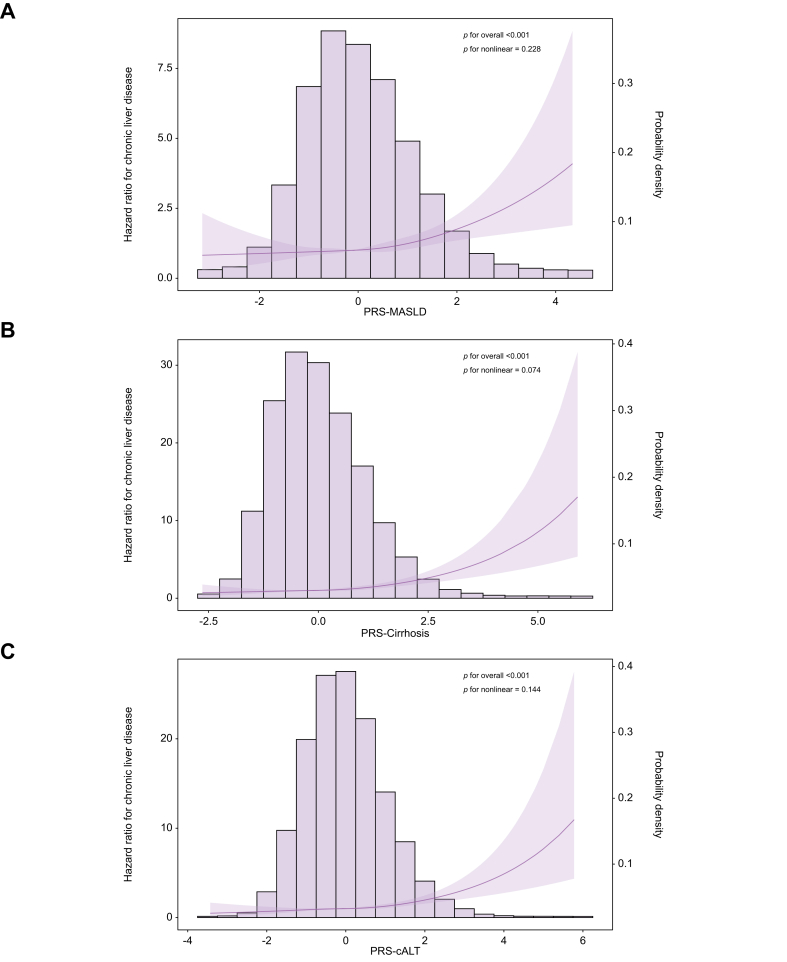
PRS and incident CLD in the MDCS (N = 26,965). (A-C) The distribution (histograms) of participants across the different PRSs and their association with incident CLD using a Cox regression model adjusting for age and sex with a fitted restricted cubic spline. The solid line represents the HR and the shaded area the 95% CI. The plots show the *p* values for the overall (linear effect) and for nonlinearity. cALT, chronically elevated ALT; CLD, chronic liver disease; MASLD, metabolic dysfunction-associated steatotic liver disease; MDCS, Malmö Diet and Cancer Study; PRS, polygenic risk score.

Overall, risk of CLD increased linearly by increasing level of PRS (Fig. 4). Compared to the lowest deciles of polygenic risk, participants in the top deciles of PRS-MASLD (HR 2.98; 95% CI 1.08-8.19; *p =* 0.035), PRS-cirrhosis (HR 5.05; 95% CI 1.73-14.79; *p =* 0.0031), and PRS-cALT (HR 2.61; 95% CI 1.02-6.68; *p =* 0.045) had a higher risk of CLD (data not tabulated). The effect of individual genetic variants on CLD is shown in Table S5. Analyses confirmed an association between genetic variation in *PNPLA3, TM6SF2, SERPINA1,* and *ARHGEF28* with risk of CLD. Other genetic variants showed non-significant effects but, with a few exceptions, associations were directionally concordant with previously reported effects. The *PNPLA3* rs738409, PRS-MASLD, PRS-cirrhosis, and PRS-cALT were associated with all specific liver outcomes including SLD, cirrhosis and HCC. There was no significant heterogeneity in associations by age and sex but associations between PRS and liver outcomes tended to be stronger among those below age 60 years at baseline (Table S6).

### Combined impact of cardiometabolic, lifestyle and genetic risk factors

Compared to a model including only age, sex and educational level (C-statistic = 0.6636), adding information on cardiometabolic, lifestyle and genetic risk factors all improved prediction (Table 3). In a model including age, sex, educational level, metabolic and lifestyle risk factors the C-statistic increased to 0.7211. Additional inclusion of the *PNPLA3* rs738409 genetic variant and the different PRSs significantly improved the C-statistic, however, the absolute increases in C-statistic were very modest (Table 3). On top of cardiometabolic and lifestyle risk factors, only the PRS-cirrhosis improved the C-statistic (C-statistic = 0.7345) above that of the *PNPLA3* rs738409 genetic variant (C-statistic = 0.7301). Overall, we found little evidence for heterogeneity in associations between lifestyle risk factors and risk of CLD depending on cardiometabolic risk factors. For the association between coffee intake and CLD, however, there was some evidence suggesting that the protective effect was limited to those with pre-existing cardiometabolic risk factors (*p* interaction = 0.040; Table 2). Similarly, we observed limited evidence for heterogeneity in effects of cardiometabolic and lifestyle risk factors on CLD based on underlying genetic predisposition (Table S5).

**Table 3:** Harrell’s C (concordance) statistic for the added benefit of metabolic, lifestyle and genetic risk factors in prediction of CLD in the MDCS (N = 26,725 with complete data on all included predictors).

Model	C-statistic	*p* value (LR)
Age, sex, and educational level	0.6636	—
+ Metabolic risk factors∗	0.6968	<0.00001
+ Lifestyle risk factors∗∗	0.7017	<0.00001
+ *PNPLA3* rs738409	0.6766	0.0001
+ PRS-MASLD	0.6739	<0.00001
+ PRS-cALT	0.6816	<0.00001
+ PRS-cirrhosis	0.6830	<0.00001
Age, sex, education, metabolic and lifestyle risk factors	0.7211	—
+ *PNPLA3* rs738409	0.7301	0.0002
+ PRS-MASLD	0.7269	0.0001
+ PRS-cALT	0.7296	<0.00001
+ PRS-cirrhosis	0.7345	<0.00001

cALT, chronically elevated ALT; CLD, chronic liver disease; LR, likelihood ratio; MASLD, metabolic dysfunction-associated steatotic liver disease; MDCS, Malmö Diet and Cancer Study; PRS, polygenic risk score.

Models were compared using the LR test.

∗ Metabolic risk factors included prevalent diabetes mellitus, BMI, waist circumference, hypertension and use of lipid-lowering medications.

∗∗ Lifestyle risk factors included smoking status, alcohol consumption, physical activity, and diet risk score.

## Discussion

This large prospective study confirms the importance of cardiometabolic dysfunction in risk of CLD and importantly provides novel evidence suggesting that lifestyle risk factors increase risk of CLD independently of underlying cardiometabolic health and genetic predisposition. While our results suggest that novel PRSs for MASLD and cirrhosis are strongly associated with higher risk of CLD, they appear to have limited utility for prediction of CLD on top of cardiometabolic and lifestyle risk factors in the general population.

We confirmed previously known associations between several cardiometabolic risk factors and risk of CLD in the general population. Diabetes mellitus was associated with a more than two-fold increased risk of CLD, which is in line with previously reported risk estimates.^21^^,^^22^ There appear to be complex bidirectional pathways between MASLD, type 2 diabetes and obesity. A Mendelian randomization study, using genetic instruments to avoid issues of confounding and reverse causation, suggested that while genetically driven type 2 diabetes, obesity and central obesity increase the risk of MASLD, MASLD also promotes development of type 2 diabetes and central obesity.^23^ In line with previous studies,^24^ we also showed that, compared to BMI, waist circumference was a stronger predictor of CLD. A recent Mendelian randomization analysis suggested that waist circumference causally increases the risk of MASLD after adjusting for BMI, while BMI was not associated with MASLD after adjusting for waist circumference.^25^ Among the other cardiometabolic traits examined in our study only HOMA-IR showed a significant and independent association with increased risk of CLD. We found no support for an association between dyslipidemia or the use of lipid-lowering drugs with CLD, which is concordant with a recent Mendelian randomization analysis that did not support dyslipidemia as a causal risk factor for MASLD.^26^ Arterial hypertension has previously been linked to risk of severe liver-related outcomes.[27], [28], [29], [30] Recent studies suggested that both measured and genetically elevated blood pressure increase the risk of liver disease.^31^^,^^32^ While we observed that hypertension was associated with a higher risk of CLD, the association was not significant after adjustment for other cardiometabolic risk factors including diabetes and adiposity.

Low educational level or socioeconomic status has previously been linked to increased risk of MASLD as well as HCC,[33], [34], [35], [36] a finding that was confirmed in our study. Most importantly, we found that an overall unhealthy lifestyle was associated with a three- to four-fold increased risk of CLD independently of educational level and cardiometabolic dysfunction. In our cohort, approximately 4% were classified as high alcohol consumers (>40 g/day for men and >30 g/day for women), and high compared to low alcohol intake was associated with a 78% increased risk of CLD. Underreporting of alcohol intake may however bias this association towards the null. A recent review concluded that smoking is associated with both development and progression of liver disease.^37^ While the observed increased risk associated with smoking in our study is significant on the population level it may hold less impact on the individual level and advice on smoking cessation should continue to emphasize other important health benefits.^37^ Participants in the upper three quartiles of physical activity compared to the lowest quartile had a lower risk of CLD, suggesting a potential threshold level above which no additional benefit is observed. The absence of a clear dose-response association may also reflect the use of self-reported physical activity. In a study from the UK Biobank using accelerometer-derived physical activity level, participants with high physical activity had a dose-dependent lower risk of overall CLD as well as MASLD.^38^

Studies examining the role of diet in CLD compared to MASLD are generally scarce and hampered by small sample sizes, with substantial heterogeneity between studies. A meta-analysis of the role of dietary patterns in MASLD suggested that Western dietary patterns (typically high in red/processed meat and refined grains) increased risk of MASLD while more health-conscious food patterns (*e.g*., Mediterranean-type dietary patterns) decreased risk of MASLD.^39^ Similar findings were observed in the UK Biobank, suggesting that high consumption of red meat and lower consumption of fruits, cereals, and dietary fiber are associated with higher risk of MASLD, cirrhosis and HCC.^40^ We found suggestive protective effects of dietary fiber and coffee intake while higher intake of red and processed meat was associated with higher risk of CLD in our cohort. In an umbrella review, a benefit of coffee consumption on liver fibrosis was seen among patients with established MASLD, but no effect on the incidence of MASLD was observed.^41^ In the UK Biobank study, coffee drinkers had a lower risk of CLD and death from CLD, and a lower risk of HCC,^42^ which is in line with observations from our cohort. High consumption of red and processed meat has been convincingly linked to type 2 diabetes in observational studies.^43^ For the role of red and processed meat in liver disease, large prospective studies are generally lacking. Cross-sectional studies indicate an association between red and processed meats with MASLD and insulin resistance.^44^ Additional large prospective studies are needed to fully elucidate the role of specific dietary components and overall dietary patterns on the progression of SLD and incidence of CLD to better inform evidence-based dietary guidelines or guide future dietary intervention studies.

In contrast to some previous investigations,^9^^,^^14^ we found that the effects of cardiometabolic and lifestyle risk factors were largely independent of genetic predisposition as assessed by the *PNPLA3* genetic variant, the PRS-MASLD, the PRS-cirrhosis, and the PRS-cALT. However, these interaction analyses may be underpowered in our study population and are not fully comparable to previous studies due to the use of a composite CLD outcome. Interestingly, compared to the *PNPLA3* genetic variant, only the PRS-cirrhosis model added additional information for prediction of CLD in our cohort. There is an overlap in genetic variants associated with a wide range of liver-related outcomes including SLD, cirrhosis and HCC. Emdin *et al.*^9^ utilized this for their multi-trait GWAS on cirrhosis, whereas previous genetic investigations have tried to examine each etiological subtype of SLD or cirrhosis separately resulting in small sample sizes. Since pathological processes may, to a large extent, be shared regardless of underlying etiology, future GWAS could harness the potentially improved statistical power in combining several related liver outcomes in order to better identify genetic variants that predict more severe liver disease. Although all three PRSs were strongly associated with a higher risk of CLD, the added benefit for prediction was marginal.

This finding is in line with a previous investigation suggesting no benefit of adding PRSs to established risk scores, including routine biomarkers such as the aspartate aminotransferase-to-platelet ratio index and the fibrosis-4 index.^45^ A plausible explanation for the lack of benefit of including the PRSs on top of cardiometabolic and lifestyle risk factors in our study could be the pleiotropic nature of most of the included genetic variants. Several of the so far identified genetic variants for MASLD and cirrhosis are known to also affect several cardiometabolic traits.

The main strengths of this study include the use of a large population-based study with extensive data collection, including dietary assessment of high relative validity, direct measurements of anthropometrics and blood pressure, and GWAS genotyping. Further, the unique personal identification number held by all inhabitants in Sweden allows for registry linkage to assess outcomes and thereby low loss to follow-up (<0.5%, due to emigration from Sweden). The included diagnoses in the primary endpoint are likely to lead to hospitalization or death and are therefore well captured by the national patient registries in Sweden which are both validated and provide outcome data of high quality. Our findings have several important implications. First, our data suggest an important role of lifestyle risk factors for the development of CLD that is largely independent of underlying cardiometabolic risk factors and genetic predisposition. This finding has importance for the primary prevention of CLD, suggesting that public health strategies in line with current recommendations for prevention of cardiometabolic diseases will likely lower the incidence of CLD. Secondly, while great efforts have been made in recent years to highlight the polygenic nature of lifestyle-related liver disease, so far adding genetic information to better predict incident CLD has limited utility on top of established risk factors.

While overall sample size was large and follow-up time considerable, the number of CLD events was limited, which reduces statistical power to detect weak effects as well as potential multiplicative interactions. The observational nature of the study hampers causal inference and residual confounding by covariates adjusted for (due to measurement error) and unmeasured confounders may impact observed associations. This is particularly important for assessment of the role of lifestyle risk factors which are self-reported and thus subjected to both measurement error and bias. We examined the association between a select number of dietary components separately and combined this into a diet risk score. Since not all included components were associated with CLD, the overall impact of an unhealthy diet as defined in our study may thus be underestimated. In observational settings a healthy diet can be defined using different approaches. In our study we opted to focus on a smaller set of dietary factors previously known to be associated with both liver disease and cardiometabolic diseases. The use of unweighted diet and lifestyle risk scores (*i.e.* assigning equal weight to all components) will also attenuate observed associations towards the null since risk factors that confer a very high risk (*i.e*. alcohol intake) will be counted as equal to risk factors conferring a more modest effect (*i.e*. diet). However, the approach used still allows for a sufficient ranking of individuals and assigning relative weights is problematic since there is a lack of consensus regarding the proposed relative effects of the included risk factors. Unfortunately, we had no possibility to ascertain presence of hepatic steatosis or other more severe liver damage at baseline apart from pre-existing diagnoses of CLD or other liver diseases. It is however plausible that the observed associations among those with poor cardiometabolic health would be similar in a population with verified MASLD due to the expected high prevalence of steatosis in this subpopulation. A major limitation is that we also lacked data on liver enzymes and liver function and were therefore not able to examine if the examined predictors for CLD in our study add predictive value above that of established risk scores. Notably, the C-indexes of established scores typically exceed those reported in our study, which further highlights the importance of such established markers for prediction of CLD in a general population.^45^

In this comprehensive analysis of risk factors for CLD in a general population, we confirm the importance of cardiometabolic perturbations. We further validate the association between several well-known and more novel genetic variants, which have been associated with MASLD and cirrhosis, in relation to the incidence of CLD in the general population. High polygenic risk of MASLD and cirrhosis conferred an increased risk of CLD but had limited predictive capability on top of cardiometabolic and lifestyle risk factors. A healthy lifestyle that promotes cardiometabolic health is likely to be beneficial for lowering the risk of CLD in the general population, irrespective of pre-existing cardiometabolic dysfunction and genetic predisposition. Since the absolute risk of CLD is low compared to that of cardiometabolic diseases it is reassuring that the same targets for primary prevention are highly relevant for CLD.

## Abbreviations

ALD, alcohol-related liver disease; cALT, chronically elevated ALT; CLD, chronic liver disease; HOMA-IR, homeostatic model assessment for insulin resistance; HR, hazard ratio; MASLD, metabolic dysfunction-associated steatotic liver disease; MDCS, Malmö Diet and Cancer Study; MDCS-CC, Malmö Diet and Cancer Study, Cardiovascular Cohort; PRS, polygenic risk score; SD, standard deviation; SLD, steatotic liver disease

## Financial support

ID was supported by grants from the Swedish Society for Medical Research, Dr P Håkansson foundation, and the Påhlsson Foundation. The study was additionally supported by grants to MO-M from the Swedish Research Council (2021-03291), the Swedish Heart and Lung Foundation (20200711), the regional Region Skåne County Council ALF grant (2022-0258) and the Novo Nordisk Foundation (NNF20OC0063886). The funders had no role in conceptualization, design, data collection, analysis, decision to publish or preparation of the manuscript.

## Conflict of interest

The authors declare that they have no conflict of interest.

Please refer to the accompanying ICMJE disclosure forms for further details.

## Authors’ contributions

Study Concept and Design: ID. Data Analysis: ID. Manuscript Preparation: ID. Critical Manuscript Review and interpretation of results: All authors.

## Data availability statement

Datasets analyzed during the current study are not publicly available due to the nature of the sensitive personal data and study materials. However, procedures for sharing data, analytic methods, and study materials for reproducing the results following Swedish legislation can be arranged by contacting the corresponding author or study organization (https://www.malmo-kohorter.lu.se/malmo-kost-cancer-mkc).

## Ethics approval and consent to participate

The study was conducted in ethical accordance with the World Medical Association Declaration of Helsinki. The protocol was approved by the Regional Ethical Review Board in Lund, Sweden (Dnr § LU 51-90, 2007/166). Written and oral informed consent for inclusion and publication was given by all subjects prior to participation.
