# Supplementary material for: Lifestyle and genetic risk of chronic liver disease in metabolically healthy and unhealthy individuals from the general population
Source: JHEP Rep. 2024 Apr 26;6(8):101105. doi: 10.1016/j.jhepr.2024.101105 (PMC11268350; doi:10.1016/j.jhepr.2024.101105)
Supplement: Multimedia component 1 [file mmc1.pdf]

Lifestyle and genetic risk of chronic liver disease in metabolically healthy and unhealthy individuals from the general population

Isabel Drake, Alice Giontella, Mariam Miari, Kristina Önnerhag, Marju Orho-Melander

Table of contents

Supplementary Data Description……………………………………………………….…2
Supplementary Tables…………………………………………………………………...5
Supplementary References………………………………………………………………17

Supplementary Data Description

Anthropometric, cardio-metabolic and blood measurements

At MDCS baseline examinations trained nurses measured height (m) and weight (kg) and body mass index (BMI) was calculated as weight divided by height squared (kg/m^2). Waist circumference (cm) was measured midway between the lowest rib margin and iliac crest. Blood pressure (mmHg) was measured using a mercury-column phygomanometer after 5 min of supine rest. Hypertension was defined as blood pressure >130/85 mmHg and/or use of anti-hypertensive medication(s). Prevalent diabetes mellitus at baseline was based on self-reported history of diabetes, diabetes diagnosis in national/local registries, current use of diabetes medications or fasting whole blood glucose of at least 6.1 mmol/l (corresponding to plasma glucose ≥7.0 mmol/L) at baseline examination.

All fasting blood samples were donated after an overnight fast and stored at −80°C. Fasting glucose, fasting insulin, high-density lipoprotein (HDL, mmol/l), and triglycerides (mmol/l) were measured at the Department of Clinical Chemistry, Skåne University Hospital in Malmö, which is attached to a national standardization system. Low-density lipoprotein (LDL) was estimated using Friedewald's formula. Fasting glucose at baseline was measured in whole blood by a hexokinase-glucose-6-phosphate dehydrogenase method (1). A constant factor of 1·11 was used to convert concentration in whole blood to the equivalent concentration in plasma (2). Homeostatic Model Assessment – Insulin Resistance (HOMA-IR) was calculated according to Matthews et al. (3) by using the formula: (fasting insulin × fasting glucose)/22.5, where insulin is expressed as mIU/l and glucose as mmol/l (1). C-reactive protein (CRP) concentration using the high-sensitive C-reactive protein (hsCRP) test, was performed using the Tina-quant® CRP latex assay (Roche Diagnostics, Basel, Switzerland) on an ADVIA® 1650 Chemistry System (Bayer healthcare, NY, USA).

Lifestyle variables

Age and sex were extracted from the participants' Swedish personal identification number. Educational level was categorized based on years and level of education completed i.e., less than 9 years or completed elementary school, middle school, high school or at least one year of studies at advanced level after high school but without degree, or university degree.

Smoking status was categorized as never, former or current (including irregular) based on self-reported use in the baseline questionnaire. Alcohol consumption was estimated based on information from both the baseline questionnaire and the reported intake during a 7-day food record that included detailed registration of cooked meals, medications, supplements and cold beverages (4). Non-consumers of alcohol (i.e., defined as those reporting no alcohol intake during the preceding year in the baseline questionnaire and reporting no intake during the 7-day registration) were classified as zero consumers while alcohol intake among consumers was categorized as low, moderate or high (i.e. <15, 15–30, or >30 g/day for women, and <20, 20–40, or >40 g/day for men). Level of leisure-time physical activity was assessed by participants reporting reported the number of minutes per week for seventeen different leisure-time activities and combined into a physical activity score (5). Participants were ranked from low to high leisure-time physical activity level by dividing them into sex-specific quartiles of total score.

Dietary intakes were assessed using a modified diet history method combining the 7-day food record with a 196-item semi-quantitative food questionnaire. Overall eating habits, quality of reported intakes in the food record and the questionnaire and potential overlap using the two modalities were further assessed using a 45-60 minutes dietary interview with a trained nutritionist (4). Reported food intake was used to calculate total dietary fiber intake using the food and nutrient data base from the Swedish National Food Agency (4). The reproducibility and validity of the diet assessment method has been described previously (6-8). We examined three previously proposed 'healthy' dietary components (dietary fiber, fruits and vegetables, and coffee) and two 'unhealthy' components (sugar-sweetened beverages and red and processed meat) dietary components. Dietary components examined were selected based on the previously reported directionally consistent associations with both liver-related outcomes and cardiometabolic diseases (9-12). Selection was further guided based on availability of data in the MDCS. Dietary intakes were energy-adjusted by calculating the relative intake in grams per 1000 kcal of estimated total energy intake.

Supplementary Tables

Table S1 ICD-codes and number of prevalent and incident first events of chronic liver disease (CLD) in the Malmö Diet and Cancer Study (N=30,446) identified in Swedish national registries including the inpatient register, hospital-based outpatient care and cause-of-death register. Only the first recorded event of the included endpoints is shown. Subjects with an incident diagnosis of chronic viral hepatitis and/or other specified cause of liver disease (n=82) were not included (ICD-10 B18, B19, E83-0, E83.1, K71, K74.3, K74.5, K75.2, K75.3, K75.4, K75.8, K75.9).

Diagnosis	ICD-10 codes	$n_{incident}$	ICD-9 codes	$n_{incident}$	$n_{prevalent}$	Total $n_{incident}$ (ICD-9 + 10 codes)
Acute and subacute liver failure	K72.0	52	570	6	4	58
Chronic liver failure	K72.1	2	572.8	1	0	3
Liver failure	K72.9, K70.4,	116	-	-	-	116
Cirrhosis	K74.6, K70.3,	112	571.5	7	3	119
Portal hypertension	K76.6	6	572.3	2	0	8
Hepatorenal syndrome	K76.7	3	572.4	2	0	5
Esophageal varices	I85.0, I85.9	38	456.0, 456.20, 456.1, 456.21	11	10	49
Ascites*	R18.9, TJA10	32	789.5	0	3	32
Liver encephalopathy	-	-	572.2	0	0	1
Hepatocellular carcinoma	C22.0	48	155	12	2	75
Liver transplantation	JJC00, JJC10, JJC20, DJ005, DJ006, JJC30, JJC40	0	5051, 5059	0	0	0
**Total CLD**		410		41	22	451***

* Only ascites cases with a subsequent additional diagnosis of CLD were included.
** Of the total number of incident events, 82 cases (18.2%) were identified in the cause-of-death registry.

Table S2 List of genetic variants and weights used to construct the weighted polygenic risk scores (PRS) for MASLD (PRS-MASLD), cALT (PRS-cALT), and liver cirrhosis (PRS-cirrhosis).

PRS-MASLD				
Variant	Gene	Minor allele	MAF	Weight[a]
rs738408	*PNPLA3*	T	0.2098	32.721
rs8107974	*TM6SF2*	T	0.1033	22.859
rs2642442	*MARC1*	C	0.2987	-7.930
rs7029757	*TOR1B*	A	0.0902	-6,409
rs429358	*APOE*	C	0.1640	-12,374
rs10787429	*GPAM*	T	0.2838	8.656
rs140201358	*PNPLA2*	G	0.0174	5.480
rs62033400	*FTO*	G	0.4104	5.493
rs9303144	*SREBF1*	C	0.3001	5.675
rs626283	*TMC4/MBOAT7*	C	0.4256	6.15
rs8113542	*INSR*	G	0.2494	5.531
rs79953491	*COBLL1*	G	0.1245	-5.894
rs4665972	*GCKR*	T	0.3713	10.681
rs1229984	*ADH1B*	T	0.0215	-6.538
rs4423880*	*MTTP*	A	0.2576	-6.615
rs112875651	*TRIB1*	A	0.4002	-9.344
PRS-cirrhosis				
Variant	Gene	Minor allele	MAF	Weight[b]
rs738409	*PNPLA3*	G	0.2098	0.4886
rs58542926	*TM6SF2*	T	0.1028	0.3646
rs2642438	*MARC1*	A	0.2749	-0.0943
rs7029757	*TOR1B*	A	0.0902	-0.1625
rs429358	*APOE*	C	0.1640	-0.1625
rs28929474	*SERPINA1*	T	0.0286	0.7080
rs6834314	*HSD17B13*	G	0.3045	-0.1625
rs12904	*EFNA1*	A	0.4294	-0.1054
rs888655	*ARHGEF28*	A	0.2793	-0.0726
rs9398804	*CENPW*	A	0.4312	-0.0726
rs1006195**	*HMBS*	T	0.3829	0.2070
rs1883711	*MAFB*	C	0.0662	0.1906
PRS-cALT				
Variant	*Gene*	Minor allele	MAF	Weight[c]
rs738408	*PNPLA3*	T	0.2098	0.269
rs2642438	*MTARC1*	A	0.2752	-0.079
rs6734238	*IL1RN*	G	0.395	-0.059
rs13389219	*COBLL1; SCN2A*	T	0.424	-0.050
rs17036160	*PPARG*	T	0.1423	-0.073
rs10433937	*HSD17B13*	G	0.3039	-0.084
rs17598226	*MTTP*	G	0.2583	-0.041
rs4841132	*PPP1R3B*	A	0.0947	0.130
rs2980888	*lnc-TRIB1; WASHC5*	T	0.2696	0.139
rs10883451	*ERLIN1*	C	0.4695	-0.161
rs4918722	*GPAM*	C	0.285	0.075
rs28929474	*SERPINA1*	T	0.02876	0.481
rs56094641	*FTO*	G	0.4205	0.040
rs1801689	*APOH*	C	0.02567	0.176
rs11668950	*IFI30;MPV17L2;PIK3R*	A	0.2247	0.041
rs58542926	*TM6SF2*	T	0.1028	0.222

rs5117	*APOE;APOC1*	C	0.2422	-0.080

[a] Weights are the Z-scores from the GOLDPlus European ancestry meta-analysis presented in Chen et al. Nature Genetics 2023 (DOI: 10.1038/s41588-023-01497-6) (13). Negative weights were used if the reported risk-increasing allele was different from the minor allele.

[b] Weights are the natural log of odds ratios for liver cirrhosis from Emdin et al. Gastroenterology 2021 (DOI: 10.1053/j.gastro.2020.12.011) (14). Negative weights were used if the reported risk-increasing allele was different from the minor allele.

[c] Weights are the beta coefficients for unexplained chronically elevated ALT levels as a proxy for MASLD in Vojkuvic et al. Nature Genetics 2022 (DOI: 10.1038/s41588-022-01078-z) (15). Negative weights were used if the reported risk-increasing allele was different from the minor allele.

* Proxy variant for rs138764179 identified by Chen et al. (13)

** Proxy variant for rs1799992 identified by Emdin et al. (14)

Table S3 Cardiometabolic risk factors for chronic liver disease (CLD) in the Malmö Diet and Cancer Study (MDCS; *n*=27,991) and the sub-sample with fasting blood samples taken at baseline (*n*=4,549).

Risk factor	Model*	HR	95% CI	*p* value
Prevalent diabetes mellitus (yes/no)	Model 1	2.53	1.79-3.57	1.4×10^{-7}
	Model 2	2.22	1.56-3.15	8.3×10^{-6}
Hypertension (yes/no)	Model 1	1.30	1.05-1.61	1.4×10^{-2}
	Model 2	1.15	0.92-1.43	0.21
Lipid-lowering drugs (yes/no)	Model 1	1.23	0.74-2.04	0.42
	Model 2	1.10	0.66-1.82	0.72
Body mass index, per SD increase	Model 1	1.32	1.19-1.46	2.3×10^{-7}
	Model 2	1.26	1.13-1.40	2.6×10^{-5}
Waist circumference, per SD increase	Model 1	1.59	1.40-1.81	4.6×10^{-13}
	Model 2	1.93	1.49-2.50	6.3×10^{-7}
	Sub-sample only			
Fasting glucose, per SD increase	Model 1	1.64	0.88-3.08	0.12
	Model 2	1.43	0.74-2.74	0.29
HbA1c, per SD increase	Model 1	1.14	0.72-1.80	0.58
	Model 2	1.10	0.69-1.74	0.69
HOMA-IR, per SD increase	Model 1	2.02	1.58-2.58	1.8×10^{-8}
	Model 2	2.11	1.62-2.75	2.8×10^{-8}
LDL, per SD increase	Model 1	0.86	0.63-1.16	0.31
	Model 2	0.83	0.61-1.13	0.23
HDL, per SD increase	Model 1	0.84	0.60-1.18	0.32
	Model 2	0.91	0.64-1.30	0.61
Triglycerides, per SD increase	Model 1	1.34	0.98-1.81	0.063
	Model 2	1.26	0.91-1.73	0.17
hsCRP, per SD increase	Model 1	1.35	1.01-1.82	0.046
	Model 2	1.28	0.94-1.75	0.12

* Model 1 presents hazard ratios (HR) and 95% confidence intervals from a Cox proportional hazards regression model adjusting for age and sex. Model 2 includes adjustment for age, sex, prevalent diabetes mellitus, body mass index, hypertension and use of lipid-lowering drugs.

Table S4 Multiplicative interaction terms between cardiometabolic, lifestyle and genetic risk factors on risk of chronic liver disease from a Cox proportional hazards regression model with adjustment for age, sex, and educational level. Nominally significant interaction terms (p<0.05) are marked in bold font.

Risk factors	*PNPLA3* rs738409	PRS-MASLD	PRS-cirrhosis	PRS-cALT
**Metabolic health status**	**0.85 (0.75-0.97)***	1.00 (0.92-1.08)	0.97 (0.90-1.04)	1.00 (0.92-1.08)
Prevalent diabetes mellitus	**0.54 (0.30-0.99)***	1.01 (0.73-1.40)	1.06 (0.77-1.45)	1.25 (0.91-1.70)
Body mass index	**0.81 (0.69-0.96)***	0.99 (0.89-1.10)	1.00 (0.91-1.09)	1.02 (0.93-1.13)
Waist circumference	0.89 (0.76-1.05)	1.05 (0.94-1.16)	1.05 (0.95-1.16)	1.05 (0.95-1.16)
Hypertension	0.79 (0.57-1.08)	0.98 (0.79-1.20)	0.90 (0.74-1.09)	0.96 (0.79-1.17)
Use of lipid-lowering drugs	0.84 (0.37-1.90)	0.86 (0.52-1.44)	0.83 (0.50-1.37)	0.84 (0.51-1.39)
**Lifestyle risk score**	1.06 (0.82-1.39)	1.07 (0.90-1.26)	0.94 (0.80-1.10)	0.97 (0.82-1.14)
Smoking status	1.15 (0.95-1.39)	1.09 (0.97-1.23)	1.01 (0.90-1.13)	0.99 (0.89-1.11)
Alcohol consumption	1.09 (0.95-1.26)	1.02 (0.93-1.12)	1.02 (0.93-1.11)	1.01 (0.93-1.11)
Physical activity	0.91 (0.79-1.04)	1.00 (0.91-1.09)	1.00 (0.92-1.09)	0.95 (0.87-1.03)
Diet risk score	1.22 (0.92-1.62)	**1.25 (1.04-1.49)***	1.05 (0.88-1.24)	1.14 (0.96-1.36)
Dietary fiber	**0.83 (0.71-0.97)***	0.91 (0.82-1.00)	0.93 (0.85-1.03)	1.01 (0.92-1.11)
Fruit and vegetables	0.92 (0.81-1.06)	0.98 (0.89-1.08)	1.00 (0.92-1.09)	1.05 (0.96-1.14)
SSB	1.00 (0.86-1.17)	**1.12 (1.02-1.24)***	1.08 (0.98-1.19)	1.08 (0.98-1.18)
Coffee	0.98 (0.85-1.14)	0.98 (0.89-1.07)	1.00 (0.92-1.09)	0.96 (0.88-1.05)
Red/processed meat	0.95 (0.80-1.13)	1.03 (0.92-1.15)	0.97 (0.87-1.08)	1.01 (0.90-1.13)

* $p<0.05$

Table S5 Effect of genetic risk variants (single nucleotide polymorphism; SNP) included in polygenic risk scores on risk of chronic liver disease (CLD) in the MDCS (N=26,965). Hazard ratios (HR) and 95% confidence intervals (CI) from a Cox proportional hazards regression model adjusting for age and sex.

Gene	SNP	PRS	Genotype	HR (95% CI)	*p* value
PNPLA3	rs738409	Cirrhosis	CC	1.00 (ref)	
			CG	1.20 (0.97-1.50)	0.099
			GG	2.31 (1.61-3.32)	6.3×10^{-6}
			Per allele effect	1.38 (1.17-1.63)	1.0×10^{-4}
PNPLA3	rs738408	MASLD, cALT	CC	1.00 (ref)	
			CT	1.20 (0.97-1.50)	0.099
			TT	2.31 (1.61-3.32)	6.3×10^{-6}
			Per allele effect	1.38 (1.17-1.63)	1.0×10^{-4}
TM6SF2	rs58542926	Cirrhosis, cALT	CC	1.00 (ref)	
			CT	1.16 (0.90-1.49)	0.26
			TT	3.19 (1.75-5.83)	1.6×10^{-4}
			Per allele effect	1.34 (1.08-1.66)	8.3×10^{-3}
TM6SF2	rs8107974	MASLD	AA	1.00 (ref)	
			AT	1.15 (0.90-1.49)	0.27
			TT	3.09 (1.69-5.64)	2.4×10^{-4}
			Per allele effect	1.33 (1.07-1.64)	9.7×10^{-3}
GCKR	rs4665972	MASLD	CC	1.00 (ref)	
			CT	0.93 (0.75-1.15)	0.5
			TT	0.82 (0.59-1.16)	0.26
			Per allele effect	0.91 (0.78-1.06)	0.25
TMC4/MBOAT7	rs626283	MASLD	GG	1.00 (ref)	
			GC	1.02 (0.81-1.29)	0.84
			CC	1.15 (0.86-1.54)	0.34
			Per allele effect	1.07 (0.92-1.24)	0.38
SERPINA1	rs28929474	Cirrhosis, cALT	CC	1.00 (ref)	
			CT	1.71 (1.20-2.43)	2.9×10^{-3}
			TT	9.51 (2.37-38.20)	1.5×10^{-3}
			Per allele effect	1.85 (1.33-2.56)	2.6×10^{-4}
HSD17B13	rs6834314	Cirrhosis	AA	1.00 (ref)	
			AG	0.85 (0.68-1.05)	0.13
			GG	0.75 (0.51-1.11)	0.15
			Per allele effect	0.86 (0.73-1.01)	0.065
HSD17B13	rs10433937	cALT	TT	1.00 (ref)	
			TG	0.84 (0.68-1.05)	0.13

			GG	0.73 (0.49-1.08)	0.12
			Per allele effect	0.85 (0.72-1.00)	0.049
MARC_1	rs2642438	Cirrhosis, cALT	GG	1.00 (ref)	
			GA	0.97 (0.78-1.20)	0.79
			AA	0.78 (0.51-1.21)	0.27
			Per allele effect	0.93 (0.79-1.09)	0.36
MARC_1	rs2642442	MASLD	TT	1.00 (ref)	
			TC	0.92 (0.74-1.14)	0.47
			CC	0.83 (0.56-1.22)	0.34
			Per allele effect	0.92 (0.78-1.07)	0.28
EFNA1	rs12904	Cirrhosis	GG	1.00 (ref)	
			GA	0.86 (0.69-1.08)	0.2
			AA	0.72 (0.52-0.98)	0.036
			Per allele effect	0.85 (0.73-0.99)	0.031
ARHGEF28	rs888655	Cirrhosis	GG	1.00 (ref)	
			AG	0.92 (0.74-1.13)	0.43
			AA	0.51 (0.30-0.84)	8.7×10^{-3}
			Per allele effect	0.82 (0.69-0.97)	0.021
CENPW	rs9398804	Cirrhosis	TT	1.00 (ref)	
			TA	1.17 (0.92-1.48)	0.21
			AA	1.12 (0.82-1.51)	0.48
			Per allele effect	1.07 (0.92-1.24)	0.38
TOR1B	rs7029757	MASLD	GG	1.00 (ref)	
			GA	1.04 (0.79-1.36)	0.8
			AA	0.38 (0.05-2.73)	0.34
			Per allele effect	0.98 (0.76-1.27)	0.89
HMBS	rs1006195	Cirrhosis	GG	1.00 (ref)	
			GT	1.17 (0.93-1.47)	0.18
			TT	1.35 (0.99-1.82)	0.055
			Per allele effect	1.16 (1.00-1.35)	0.046
APOE	rs429358	MASLD, Cirrhosis	TT	1.00 (ref)	
			TC	0.88 (0.69-1.12)	0.28
			CC	1.03 (0.55-1.94)	0.92
			Per allele effect	0.92 (0.75-1.13)	0.42
APOE; APOC1	rs5117	cALT	TT	1.00 (ref)	
			TC	1.00 (0.81-1.25)	0.91
			CC	0.73 (0.44-1.22)	0.23
			Per allele effect	0.94 (0.79-1.11)	0.46
APOH	rs1801689	cALT	AA	1.00 (ref)	

			AC	1.32 (0.86-2.01)	0.2
			CC	8.41 (2.09-33.80)	2.7 x 10^{-3}
			Per allele effect	1.49 (1.02-2.19)	0.041
MAFB	rs1883711	Cirrhosis	GG	1.00 (ref)	
			GC	0.78 (0.56-1.10)	0.16
			CC	0.58 (0.08-4.13)	0.59
			Per allele effect	0.78 (0.57-1.08)	0.13
GPAM	rs10787429	MASLD	CC	1.00 (ref)	
			CT	1.13 (0.91-1.40)	0.26
			TT	1.04 (0.70-1.53)	0.86
			Per allele effect	1.06 (0.91-1.24)	0.45
GPAM	rs4918722	cALT	TT	1.00 (ref)	
			TC	1.15 (0.92-1.42)	0.21
			CC	1.00 (0.67-1.48)	0.98
			Per allele effect	1.06 (0.90-1.24)	0.49
PNPLA2	rs140201358	MASLD	CC	1.00 (ref)	
			CG/GG*	0.85 (0.47-1.55)	0.6
			Per allele effect	0.85 (0.47-1.54)	0.59
FTO	rs62033400	MASLD	AA	1.00 (ref)	
			AG	1.08 (0.86-1.36)	0.5
			GG	1.08 (0.80-1.47)	0.61
			Per allele effect	1.05 (0.90-1.21)	0.54
FTO	rs56094641	cALT	AA	1.00 (ref)	
			AG	1.08 (0.86-1.37)	0.5
			GG	1.08 (0.80-1.46)	0.62
			Per allele effect	1.05 (0.90-1.21)	0.55
SREBF1	rs9303144	MASLD	TT	1.00 (ref)	
			TC	0.99 (0.80-1.23)	0.96
			CC	1.04 (0.72-1.51)	0.84
			Per allele effect	1.01 (0.86-1.18)	0.91
INSR	rs8113542	MASLD	AA	1.00 (ref)	
			AG	1.06 (0.86-1.32)	0.58
			GG	0.92 (0.58-1.45)	0.71
			Per allele effect	1.01 (0.86-1.20)	0.89
COBLL1	rs79953491	MASLD	AA	1.00 (ref)	
			AG	1.12 (0.88-1.43)	0.35
			GG	0.53 (0.17-1.66)	0.28
			Per allele effect	1.03 (0.83-1.28)	0.78
COBLL1; SCN2A	rs13389219	cALT	CC	1.00 (ref)	

			CT	0.94 (0.75-1.17)	0.56
			TT	0.81 (0.59-1.10)	0.18
			Per allele effect	0.91 (0.78-1.05)	0.19
ADH1B	rs1229984*	MASLD	CC	1.00 (ref)	
			CT/TT	0.63 (0.33-1.23)	0.17
			Per allele effect	0.63 (0.33-1.19)	0.16
MTTP	rs4423880	MASLD	GG	1.00 (ref)	
			GA	1.09 (0.88-1.35)	0.45
			AA	1.08 (0.71-1.63)	0.73
			Per allele effect	1.06 (0.90-1.25)	0.48
MTTP	rs17598226	cALT	CC	1.00 (ref)	
			CG	1.05 (0.85-1.31)	0.64
			GG	1.06 (0.70-1.61)	0.78
			Per allele effect	1.04 (0.88-1.23)	0.63
TRIB1	rs112875651	MASLD	GG	1.00 (ref)	
			GA	1.11 (0.89-1.40)	0.35
			AA	0.97 (0.71-1.34)	0.87
			Per allele effect	1.01 (0.87-1.17)	0.88
IL1RN	rs6734238	cALT	AA	1.00 (ref)	
			AG	1.01 (0.80-1.27)	0.95
			GG	1.18 (0.87-1.59)	0.28
			Per allele effect	1.07 (0.92-1.24)	0.36
PPARG	rs17036160	cALT	CC	1.00 (ref)	
			CT	0.93 (0.73-1.19)	0.58
			TT	0.64 (0.26-1.55)	0.33
			Per allele effect	0.90 (0.72-1.12)	0.33
PPP1R3B	rs4841132	cALT	GG	1.00 (ref)	
			GA	1.02 (0.78-1.34)	0.86
			AA	1.15 (0.43-3.08)	0.78
			Per allele effect	1.03 (0.81-1.32)	0.79
lnc-TRIB1; WASHC5	rs2980888	cALT	CC	1.00 (ref)	
			CT	1.22 (0.99-1.51)	0.061
			TT	0.60 (0.35-1.02)	0.058
			Per allele effect	1.00 (0.85-1.17)	0.96
ERLIN1	rs10883451	cALT	TT	1.00 (ref)	
			TC	0.89 (0.70-1.13)	0.32
			CC	0.90 (0.68-1.20)	0.48
			Per allele effect	0.94 (0.82-1.09)	0.44

IFI30;MPV17L2;PIK3R	rs11668950	cALT	GG	1.00 (ref)	
			GA	1.10 (0.89-1.37)	0.38
			AA	1.00 (0.62-1.61)	0.99
			Per allele effect	1.05 (0.89-1.25)	0.55

* Homozygous carriers of the minor allele were few and therefore heterozygous and homozygous carriers of the minor allele were collapsed into one category.

Table S6 *PNPLA3* rs738409 genetic risk variant and polygenic risk scores (PRSs) for metabolic dysfunction-associated steatotic liver disease (MASLD), liver cirrhosis and unexplained chronic ALT elevation (cALT) in relation to incidence of chronic liver disease (CLD), steatotic liver disease (unspecified), liver cirrhosis (all-cause) and hepatocellular carcinoma (HCC) in the Malmö Diet and Cancer Study (N=26,965) stratified by age and sex. Hazard ratios (HR) and 95% confidence intervals (CI) per risk G-allele in *PNPLA3* rs738409 and per standard deviation increase in normalized (z-score) PRS-MASLD, PRS-cirrhosis and PRS-cALT.

	CLD (n_{cases}=365)		Steatotic liver disease (n_{cases}=76)		Liver cirrhosis (n_{cases}=173)		HCC (n_{cases}=72)	
	HR (95% CI)	*p* value	HR (95% CI)	*p* value	HR (95% CI)	*p* value	HR (95% CI)	*p* value
PNPLA3 rs738409								
All	1.38 (1.17-1.62)	1.2×10^{-4}	1.39 (0.97-1.98)	0.073	1.79 (1.43-2.24)	3.5×10^{-7}	1.93 (1.37-2.72)	1.7×10^{-4}
Men	1.34 (1.08-1.66)	7.3×10^{-3}	1.57 (0.88-2.80)	0.13	1.65 (1.24-2.20)	5.8×10^{-4}	1.86 (1.22-2.83)	3.9×10^{-3}
Women	1.43 (1.11-1.84)	6.0×10^{-3}	1.29 (0.82-2.03)	0.27	2.03 (1.41-2.91)	1.2×10^{-4}	2.06 (1.14-3.71)	0.016
Age <60 years	1.45 (1.17-1.79)	7.4×10^{-4}	1.72 (1.17-2.53)	0.0061	2.05 (1.56-2.70)	2.5×10^{-7}	1.95 (1.20-3.16)	6.6×10^{-3}
Age ≥60 years	1.31 (1.01-1.68)	0.038	0.49 (0.17-1.37)	0.17	1.38 (0.93-2.05)	0.11	1.94 (1.19-3.17)	7.9×10^{-3}
PRS-MASLD								
All	1.23 (1.11-1.35)	5.6×10^{-5}	1.25 (1.01-1.55)	0.043	1.45 (1.26-1.67)	1.5×10^{-7}	1.52 (1.24-1.90=	1.0×10^{-4}
Men	1.24 (1.10-1.41)	8.0×10^{-4}	1.23 (0.86-1.77)	0.26	1.40 (1.17-1.67)	1.9×10^{-4}	1.66 (1.28-2.15)	1.2×10^{-4}
Women	1.20 (1.03-1.40)	2.1×10^{-2}	1.26 (0.96-1.65)	0.094	1.55 (1.24-1.95)	1.6×10^{-4}	1.28 (0.87-1.89)	0.20
Age <60 years	1.29 (1.13-1.47)	1.5×10^{-4}	1.48 (1.16-1.88)	0.0013	1.62 (1.36-1.92)	4.3×10^{-8}	1.51 (1.11-2.04)	8.3×10^{-3}
Age ≥60 years	1.15 (0.99-1.34)	0.064	0.65 (0.39-1.09)	0.10	1.20 (0.94-1.52)	0.14	1.56 (1.15-2.12)	3.8×10^{-3}
PRS-cirrhosis								
All	1.36 (1.24-1.50)	8.9×10^{-11}	1.38 (1.13-1.70)	1.8×10^{-3}	1.65 (1.45-1.87)	1.5×10^{-14}	1.80 (1.49-2.18)	1.9×10^{-9}
Men	1.42 (1.26-1.60)	9.3×10^{-9}	1.14 (0.79-1.64)	0.47	1.64 (1.39-1.92)	1.5×10^{-9}	2.06 (1.65-2.56)	1.0×10^{-10}
Women	1.28 (1.10-1.49)	1.2×10^{-3}	1.53 (1.19-1.95)	7.8×10^{-4}	1.66 (1.35-2.05)	2.0×10^{-6}	1.28 (0.88-1.86)	0.20
Age <60 years	1.38 (1.22-1.56)	3.8×10^{-7}	1.53 (1.22-1.91)	2.4×10^{-4}	1.72 (1.47-2.01)	1.1×10^{-11}	1.97 (1.52-2.54)	2.7×10^{-7}
Age ≥60 years	1.34 (1.16-1.55)	4.8×10^{-5}	0.96 (0.60-1.54)	0.88	1.53 (1.23-1.90)	1.4×10^{-4}	1.63 (1.23-2.17)	7.2×10^{-4}
PRS-cALT								
All	1.34 (1.21-1.47)	2.9×10^{-9}	1.11 (0.89-1.38)	0.35	1.54 (1.35-1.76)	2.1×10^{-10}	1.59 (1.29-1.95)	9.8×10^{-6}

Men	1.38 (1.22-1.56)	3.3 x 10^{-7}	0.98 (0.67-1.43)	0.91	1.59 (1.35-1.88)	5.0 x 10^{-8}	1.77 (1.39-2.26)	4.6 x10^{-6}
Women	1.26 (1.08-1.47)	2.5 x 10^{-3}	1.19 (0.91-1.55)	0.21	1.44 (1.15-1.80)	1.4 x 10^{-3}	1.23 (0.84-1.80)	0.28
Age <60 years	1.36 (1.20-1.55)	2.3 x 10^{-6}	1.13 (0.88-1.45)	0.34	1.56 (1.32-1.84)	2.1 x 10^{-7}	1.80 (1.36-2.38)	3.7 x 10^{-5}
Age ≥60 years	1.30 (1.13-1.50)	2.9 x 10^{-4}	1.06 (0.67-1.65)	0.81	1.52 (1.22-1.89)	2.2 x 10^{-4}	1.39 (1.03-1.87)	0.031

Supplementary References

1. Hedblad B, Nilsson P, Janzon L, Berglund G. Relation between insulin resistance and carotid intima-media thickness and stenosis in non-diabetic subjects. Results from a cross-sectional study in Malmo, Sweden. Diabet Med. 2000;17(4):299-307.
2. D'Orazio P, Burnett RW, Fogh-Andersen N, Jacobs E, Kuwa K, Kulpmann WR, et al. Approved IFCC recommendation on reporting results for blood glucose: International Federation of Clinical Chemistry and Laboratory Medicine Scientific Division, Working Group on Selective Electrodes and Point-of-Care Testing (IFCC-SD-WG-SEPOCT). Clin Chem Lab Med. 2006;44(12):1486-90.
3. Matthews DR, Hosker JP, Rudenski AS, Naylor BA, Treacher DF, Turner RC. Homeostasis model assessment: insulin resistance and beta-cell function from fasting plasma glucose and insulin concentrations in man. Diabetologia. 1985;28(7):412-9.
4. Wirfalt E, Mattisson I, Johansson U, Gullberg B, Wallstrom P, Berglund G. A methodological report from the Malmo Diet and Cancer study: development and evaluation of altered routines in dietary data processing. Nutr J. 2002;1:3.
5. Mattisson I, Wirfalt E, Aronsson CA, Wallstrom P, Sonestedt E, Gullberg B, et al. Misreporting of energy: prevalence, characteristics of misreporters and influence on observed risk estimates in the Malmo Diet and Cancer cohort. Br J Nutr. 2005;94(5):832-42.
6. Elmstahl S, Gullberg B, Riboli E, Saracci R, Lindgarde F. The Malmo Food Study: the reproducibility of a novel diet history method and an extensive food frequency questionnaire. Eur J Clin Nutr. 1996;50(3):134-42.
7. Elmstahl S, Riboli E, Lindgarde F, Gullberg B, Saracci R. The Malmo Food Study: the relative validity of a modified diet history method and an extensive food frequency questionnaire for measuring food intake. Eur J Clin Nutr. 1996;50(3):143-51.
8. Riboli E, Elmstahl S, Saracci R, Gullberg B, Lindgarde F. The Malmo Food Study: validity of two dietary assessment methods for measuring nutrient intake. Int J Epidemiol. 1997;26 Suppl 1:S161-73.

9. Hassani Zadeh S, Mansoori A, Hosseinzadeh M. Relationship between dietary patterns and non-alcoholic fatty liver disease: A systematic review and meta-analysis. J Gastroenterol Hepatol 2021; 36(6):1470-1478.

10. Kennedy OJ, Fallowfield JA, Poole R, Hayes PC, Parkes J, Roderick PJ. All coffee types decrease the risk of adverse clinical outcomes in chronic liver disease: a UK Biobank study. BMC Public Health. 2021; 21(1): 970.

11. Carlström M, Larsson SC. Coffee consumption and reduced risk of developing type 2 diabetes: a systematic review with meta-analysis. Nutr Rev. 2018; 76(6):395-417.

12. Miller V, Micha R, Choi E, Karageorgou D, Webb P, Mozaffarian D. Evaluation of the Quality of Evidence of the Association of Foods and Nutrients With Cardiovascular Disease and Diabetes: A Sytematic Review. JAMA Netw Open. 2022; 5(2):e2146705.

13. Chen Y, Du X, Kuppa A, Feitosa MF, Bielak LF, O'Connell JR, et al. Genome-wide association meta-analysis identifies 17 loci associated with nonalcoholic fatty liver disease. Nat Genet. 2023; 55(10):1640-1650.

14. Emdin CA, Haas M, Ajmera V, Simon TG, Homburger J, Neben C, et al. Association of Genetic Variation With Cirrhosis: A Multi-Trait Genome-Wide Association and Gene-Environment Interaction Study. Gastroenterology. 2021;160(5):1620-33 e13.

15. Vujkovic M, Ramdas S, Lorenz KM, Guo X, Darlay R, Cordell HJ, et al. A multiancestry genome-wide association study of unexplained chronic ALT elevation as a proxy for nonalcoholic fatty liver disease with histological and radiological validation. Nat Genet. 2022; 54(6):761-771.
