# Supplementary material for: Lifestyle and genetic risk of chronic liver disease in metabolically healthy and unhealthy individuals from the general population
Source: JHEP Rep. 2024 Apr 26;6(8):101105. doi: 10.1016/j.jhepr.2024.101105 (PMC11268350; doi:10.1016/j.jhepr.2024.101105)
Supplement: Multimedia component 4 [file mmc4.pdf]

Research article

JHEP|Reports

Lifestyle and genetic risk of chronic liver disease in metabolically healthy and unhealthy individuals from the general population

Isabel Drake[1,2,*], Alice Giontella[1], Mariam Miari[1], Kristina Önnerhag[3,4], Marju Orho-Melander[1]

JHEP Reports **2024**. vol. 6 | 1–11

Check for updates

Background & Aims: It is unclear to what extent lifestyle and genetic factors affect the incidence of chronic liver disease (CLD) in the general population and if lifestyle affects CLD independently of underlying cardiometabolic perturbations and genetic predisposition.

Methods: We examined 27,991 men and women aged 44-73 years from the Malmö Diet and Cancer Study recruited between 1991-1996 and followed until the end of 2020 using registry linkage (median follow-up time 25.1 years; 382 incident first-time CLD events). Associations between cardiometabolic factors, polygenic risk scores (PRSs), and lifestyle factors in relation to CLD were examined using multivariable Cox proportional hazards regression models.

Results: The incidence of CLD increased with number of cardiometabolic risk factors (the hazard ratio per each additional cardiometabolic risk factor was 1.33; 95% CI 1.21-1.45; $p = 5.1 \times 10^{-10}$). Two novel PRSs for metabolic dysfunction-associated steatotic liver disease and a PRS for cirrhosis were associated with higher risk of CLD but provided marginal predictive utility on top of other risk factors and compared to the *PNPLA3* rs738409 genetic variant. An unhealthy lifestyle (high alcohol intake, current smoking, physical inactivity and unhealthy diet) markedly increased the risk of CLD (hazard ratio 3.97, 95% CI 2.59-6.10). Observed associations between examined lifestyle factors and CLD were largely independent of cardiometabolic perturbations and polygenic risk.

Conclusions: We confirmed the importance of cardiometabolic dysfunction in relation to risk of CLD in the general population. Lifestyle risk factors were shown to be independently associated with CLD and added predictive information on top of cardiometabolic risk factors. Information on the polygenic risk of liver disease does not currently improve the prediction of CLD in the general population.

© 2024 The Author(s). Published by Elsevier B.V. on behalf of European Association for the Study of the Liver (EASL). This is an open access article under the CC BY license (http://creativecommons.org/licenses/by/4.0/).

Introduction

Liver diseases accounts for over two million deaths per year.[1] The burden of liver diseases in Europe continues to grow, owing primarily to excessive alcohol consumption and the increasing prevalence of obesity.[2] Both obesity and excessive alcohol consumption are important causes of steatotic liver disease (SLD). The worldwide prevalence of metabolic dysfunction-associated SLD (MASLD) was recently estimated to be over 30%, with a geographical distribution that varies depending on factors such as ethnicity, genetic predisposition and lifestyle factors.[3,4] For alcohol-associated liver disease (ALD), a recent systematic review suggested a prevalence of 3.5% in the general population. However, in groups with alcohol use disorder the prevalence of ALD is approximately 51%.[5] In the new revision of the SLD nomenclature, MASLD with alcohol intake (MetALD) was defined as a specific subgroup of SLD.[3] This highlights the important notion that alcohol intake and metabolic dysfunction are rarely mutually exclusive risk factors in the clinical setting.[6]

Increased fine-tuning of the classification of heterogeneous diseases such as SLD based on disease characteristics and etiology can greatly aid the targeted treatment of established disease. However, for primary prevention such subclassification tends to be less useful since the population will always be at risk of different outcomes and the presence of multiple exposures may need joint consideration. Patients with SLD are at risk of developing chronic liver disease (CLD) including cirrhosis and hepatocellular carcinoma (HCC). Due to the poor prognosis of CLD it is important to identify useful risk markers to adequately identify risk groups that may benefit from intervention or screening.

* Corresponding author. Address: Clinical Research Center House 60 Floor 13, Jan Waldenströms gata 35, SE-205 02 Malmö, Sweden.
E-mail address: isabel.drake@med.lu.se (I. Drake).
https://doi.org/10.1016/j.jhepr.2024.101105

MASLD has until recently been considered oligogenic which is in notable contrast to other cardiometabolic diseases and traits. Previous genome-wide association studies (GWAS) have typically identified only a small subset of genetic risk variants for MASLD. However, recent efforts with increased GWAS sample sizes have expanded our understanding of the potentially polygenic nature of MASLD[7,8] and cirrhosis.[9] Several lifestyle factors have also been proposed to play a role in development and management of MASLD as well as CLD,[10–12] however, few prospective studies have examined the impact of lifestyle factors on CLD in the general population.[13] It has further been reported that individuals with genetic predisposition to liver disease are at higher risk of liver damage due to exogenous risk factors.[14]

In this study, we wanted to examine how lifestyle factors associate with CLD and specifically examine if these associations differ based on underlying cardiometabolic perturbations or genetic predisposition. We further wanted to assess the potential utility of recently identified polygenic risk scores (PRSs) for the prediction of CLD in the general population.

Materials and methods

Study population

The Malmö Diet and Cancer Study (MDCS) is a population-based prospective cohort study.[15,16] In short, during 1991-1996, all inhabitants of the city of Malmö (Southern Sweden) aged between 44-73 years were invited to join the study. In total, the MDCS recruited 30,446 men and women (participation rate approximately 40%).[17] Among the MDCS participants, 28,098 participants completed the majority of baseline examinations including a detailed dietary assessment and constitute a part of the EPIC (European Prospective Investigation into Nutrition and Cancer) cohort. Baseline examinations included direct measurements and donation of non-fasting blood samples stored in a biobank, an extensive baseline questionnaire covering lifestyle, socioeconomic and disease history, and dietary assessment using a modified diet history method.[16] Between 1991 and 1994, every other participant in the MDCS was asked to join a sub-cohort (n = 6,103) in which participants underwent additional examinations including donation of fasting blood samples.[18] A flow chart of the study population including exclusion criteria for the current study is shown in Fig. 1.

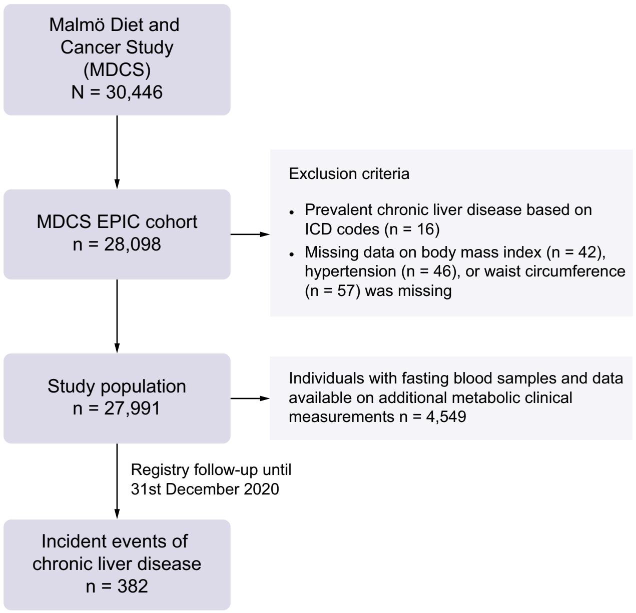

Fig. 1. Flow chart of the study population. MDCS, Malmö Diet and Cancer Study.

Ascertainment CLD at baseline and during follow-up

A composite endpoint of CLD based on ICD-9/10 codes in the National Patient Register and the Swedish Cause of Death Register was used as the primary endpoint (Table S1). Participants were followed from baseline through register linkage using their Swedish personal identification number until first incident event of CLD, emigration (<0.5%), death, or 31st December 2020. Diagnoses classified as CLD included acute and subacute liver failure (ICD-9:570 or ICD-10 K72.0), chronic liver failure (ICD-9 572.8 or ICD-10 K72.1), liver failure (ICD-10 K72.9, K70.4), cirrhosis (ICD-9 571.5 or ICD-10 K74.6, K70.3), portal hypertension (ICD-9 571.5 or ICD-10 K76.6), hepatorenal syndrome (ICD-9 572.4 or ICD-10 K76.7), esophageal varices (ICD-9 456 or ICD-10 I85.0, I85.9), ascites (ICD-9 789.5 or ICD-10 R18.9), liver encephalopathy (ICD-9 572.2), hepatocellular carcinoma (ICD-9 155 or ICD-10 C22.0), and liver transplantation (ICD-10 JJC00, JJC10, JJC20, DJ005, DJ006, JJC30, JJC40). For diagnosis of ascites, only cases with a subsequent diagnosis of another CLD event were included (n = 32), while the remainder were censored at the time of ascites diagnosis (n = 322). No participants had a prevalent diagnosis of chronic viral hepatitis and/or other non-lifestyle-related causes of liver disease at baseline (ICD-10 codes [or corresponding codes using earlier ICD-version] B18, B19, E83.0, E83.1, K71, K74.3, K74.4, K74.5, K75.2, K75.3, K75.4, K75.8, K75.9). Individuals with any incident diagnosis of chronic viral hepatitis and/or other non-lifestyle-related cause of liver disease were censored at the time of diagnosis (n = 307 of whom 82 were later diagnosed with CLD) and were not included in analysis of the primary endpoint. Sensitivity analyses were also performed to examine the association with specific liver-related outcomes including cirrhosis and HCC, as well as SLD (ICD-10 K76.0), which was not included in the primary endpoint.

Ascertainment of cardiometabolic health at baseline examinations

A detailed description of baseline assessments of anthropometric, cardiometabolic and blood measurements is provided in the Supplementary Material. Study participants were grouped according to their cardiometabolic health at baseline based on the number of fulfilled adult criteria for metabolic dysfunction as outlined in the definition of MASLD.[3,19] An unhealthy cardiometabolic status was defined as fulfilling at least one out of five criteria: 1) BMI ≥25 kg/m^2, 2) waist circumference >94 cm for men or >80 cm for women, 3) prevalent diabetes mellitus, 4) presence of hypertension (blood pressure ≥130/85 mmHg) and/or use of anti-hypertensive medication, or 5) use of lipid-lowering

medications. Among individuals with fasting blood samples taken at baseline, analyses were performed to examine additional cardiometabolic risk factors including plasma triglycerides (mmol/l), high-density lipoprotein-cholesterol (mmol/L), fasting glucose (mmol/L), homeostatic model assessment for insulin resistance (HOMA-IR), and plasma high-sensitivity C-reactive protein level (mg/L).

Lifestyle variables

A detailed description of the included lifestyle variables is found in the Supplementary Material. We examined four modifiable lifestyle factors: alcohol consumption, smoking status, physical activity and diet. Smoking status was categorized as never, former or current (including irregular). Alcohol consumption was categorized as zero, low, moderate or high. Leisure-time physical activity level was categorized by dividing participants into sex-specific quartiles of a physical activity score based on self-reported time spent on leisure-time physical activities. We examined three proposed 'healthy' dietary components (dietary fiber, fruits and vegetables, and coffee) and two 'unhealthy' components (sugar-sweetened beverages and red and processed meat). A diet risk score was constructed to reflect overall diet by dividing participants into tertiles of intakes of the five components. The score was constructed by assigning points (1, 2, or 3) based on tertiles of energy-adjusted intakes and adding the five components together into a total score (ranging from 5-15 points). Low intakes of dietary fiber, fruit and vegetables and coffee were given low points whereas low intakes of sugar-sweetened beverages and red and processed meats were given high points based on previously reported directions of effect (see Supplementary Material for more information). The total score aimed to reflect a range from unhealthy (low points) to healthy (high points) diet. To assess the combined impact of overall lifestyle we constructed a lifestyle risk score where one point each were received for current smoking, high alcohol consumption, low physical activity (quartile 1), and unhealthy diet (diet risk score 5-7 points), where 0 points reflected absence and 4 points reflected presence of all four risk factors. Participants with no lifestyle risk factors were classified as healthy whereas those with 3-4 risk factors were classified as unhealthy.

Genotyping and PRSs

Genotyping of MDCS participants was performed using the Illumina GSA v1 genotyping array and details of the genotyping and quality control procedures have been described in detail previously.[20] We considered genetic variants previously associated with MASLD and cirrhosis for construction of three PRSs. The PRS-MASLD included 16 independent genetic variants associated with MASLD in the European ancestry-only meta-analysis by Chen *et al.*.[8] The PRS-cALT included 17 genetic variants identified using unexplained chronically elevated ALT (cALT) levels as a proxy for MASLD that showed concordant effect estimates in cohorts with imaging or histology verified MASLD in a study by Vojkovic *et al.*.[7] The PRS-cirrhosis included 12 genetic variants associated with cirrhosis in a study by Emdin *et al.*.[9] Genetic variants in four genes (*PNPLA3, TM6SF2, MARC1* and *APOE*) were included in all three scores. Genetic variants in two additional genes (*TOR1B* and *SERPINA1*) were included in two of the scores whereas the remaining variants were specific to the respective PRSs. Genotypes of two variants, MTTP rs138765179 in the PRS-MASLD score and HMBS rs1799992 in the PRS-cirrhosis score, were not available and we therefore used two proxy variants ($r^{2=}1$ and D'=1). A list of all included variants as well as weights used to construct the PRSs are shown in Table S2. All genetic variants were coded as 0, 1 or 2 for non-carriers, heterozygous carriers, and homozygous carriers of the minor allele, respectively. The PRSs were calculated by summing the number of minor alleles and weighting by their corresponding effect sizes (reported z-scores, natural log odds ratios or beta coefficients). The effect of the individual genetic variants on CLD by genotype and the per minor allele effects were also examined.

Statistical analysis

Baseline characteristics of the study population and differences by cardiometabolic health status at baseline were assessed using the t-test or the Kruskal-Wallis test for continuous variables and the chi-square test for categorical variables. The cumulative incidence of CLD by number of fulfilled cardiometabolic criteria was estimated using a competing risk regression model based on the Fine and Gray method taking into account the competing risk of non-CLD deaths, and with adjustment for age and sex. A Cox proportional hazards regression model with follow-up time as the underlying time-metric was used to assess hazard ratios (HRs) and 95% CIs for CLD by differences in baseline cardiometabolic, lifestyle and genetic risk factors. An age- and sex-adjusted Cox proportional hazards model was used as a basic model to assess the role of each risk factor individually, and a mutually adjusted model including all lifestyle risk factors simultaneously was constructed to identify lifestyle risk factors independently associated with CLD. The cumulative incidence of CLD by the lifestyle risk score (healthy, moderate, unhealthy) was estimated using a competing risk regression model with adjustment for age, sex, and educational level. To examine the association between PRS-MASLD, PRS-cirrhosis and PRS-cALT with CLD, we fitted restricted cubic splines to Cox regression models adjusting for age and sex and assessed the HRs and 95% CIs per standard deviation (SD) increase. Heterogeneity in the associations between individual lifestyle risk factors in relation to CLD by number of cardiometabolic risk factors at baseline was examined by including the multiplicative interaction terms in the age- and sex-adjusted models and in the fully adjusted models. We further assessed multiplicative interactions between cardiometabolic and lifestyle risk factors with the *PNPLA3* rs738409 genetic variant, PRS-MASLD, PRS-cirrhosis and PRS-cALT in Cox regression models adjusting for age, sex, and educational level. To examine the predictive utility of cardiometabolic, lifestyle and genetic risk factors for CLD we calculated the Harrell's C-statistic and used the likelihood ratio test to assess significant model improvement. All multivariable analyses were complete case analyses and thus total number differed slightly between analyses due to missing data on some covariates, with sample sizes ranging from 27,991 to 26,725 in analyses that included all covariates. Covariates with missing data included educational level (n = 70), smoking status (n = 12), physical activity (n = 186) and genotype data (n = 1025). As a sensitivity analysis, we examined associations with specific liver-related outcomes including cirrhosis, HCC and SLD. Deviation from the proportional hazards assumption in all Cox regression models was tested using the Schoenfeld test; no

significant deviations were noted (all p >0.10). Non-linear effects by non-categorized continuous variables were tested by fitting restricted cubic splines and the likelihood ratio test was used to test for significant deviations from linearity; no significant deviations were noted (all p >0.10). Continuous variables with a skewed distribution were transformed using a natural log transformation and standardized to a normal distribution with mean 0 and SD of 1 and the effects per 1 SD increase were estimated. All tests were two-sided and p values <0.05 were considered statistically significant. All analyses were performed in Stata/SE Version 15.1 and R version 4.3.1 (The R Foundation for Statistical Computing Platform).

Results

Description of study population

During a median follow-up time of 25.1 years (IQR 18.6-27.0 years), 382 incident events of CLD occurred. Baseline characteristics of participants are shown in Table 1. In the study population, 4.5% had diabetes mellitus at baseline and mean BMI was 25.7 kg/m^2 (SD = 4.0). At baseline, 55% were defined as hypertensive and 3.2% reported current use of lipid-lowering drugs (Table 1). In total 75.7% of the study population fulfilled at least one criteria of metabolic dysfunction (27.9% fulfilled one criterium only, 21.8% fulfilled two criteria, and 26.0% fulfilled three or more criteria).

Cardiometabolic risk factors

The cumulative incidence of CLD increased by number of fulfilled cardiometabolic criteria (Fig. 2). After adjustment for age, sex, and educational level, the risk increase per number of fulfilled criteria was 1.33 (95% CI 1.21-1.45; $p = 5.1 \times 10^{-10}$) and individuals fulfilling all five cardiometabolic criteria had an 8-fold higher risk of CLD compared to those with none (HR 8.33; 95% CI 3.02-23.03; $p = 4.4 \times 10^{-5}$). The effect of individual cardiometabolic risk factors on risk of CLD was examined after

Table 1. Baseline lifestyle, genetic and metabolic characteristics of the MDCS (N = 27,991) and the MDCS-CC (N = 4,549) overall and by categorization of participants based on metabolic health status at baseline.

Characteristic	All participants	Metabolically healthy	Metabolically unhealthy	*p* value*
Number of participants, n (%)	27,991	6,817 (24.4)	21,174 (75.7)	—
Number of incident CLD cases, n (%)	382 (1.4)	58 (0.9)	324 (1.5)	<0.0001
Age, years (SD)	58.1 (7.6)	55.3 (7.2)	59.0 (7.5)	<0.0001
Male sex, n (%)	11,020 (39.4)	1,772 (26.0)	9,248 (43.7)	<0.0001
Prevalent diabetes mellitus, n (%)	1,245 (4.5)	0 (0)	1,245 (5.9)	<0.0001
Body mass index, kg/m^2	25.7 (4.0)	22.3 (1.8)	26.9 (3.8)	<0.0001
Waist circumference, cm (SD)	84.1 (12.9)	73.8 (7.9)	87.4 (12.5)	<0.0001
Hypertension, n (%)	15,383 (55.0)	0 (0)	15,383 (72.7)	<0.0001
Use of lipid-lowering drugs, n (%)	903 (3.2)	0 (0)	903 (4.3)	<0.0001
Educational level, n (%)				<0.0001
Elementary school	11,731 (42.0)	2,116 (31.1)	9,615 (45.5)	
Middle school	7,299 (26.1)	1,916 (28.2)	5,383 (25.5)	
High school	4,924 (17.6)	1,374 (20.2)	3,550 (16.8)	
University degree	3,967 (14.2)	1,399 (20.6)	2,568 (12.2)	
Smoking status, n (%)				<0.0001
Never	10,612 (37.9)	2,467 (36.2)	8,145 (38.5)	
Former	9,461 (33.8)	1,986 (29.1)	7,475 (35.3)	
Current	7,906 (28.3)	2,363 (34.7)	5,543 (26.2)	
Alcohol consumption, n (%)				<0.0001
Zero	1,699 (6.1)	333 (4.9)	1,366 (6.5)	
Low	20,308 (72.6)	5,026 (73.7)	15,282 (72.2)	
Moderate	4,780 (17.1)	1,216 (17.8)	3,564 (16.8)	
High	1,204 (4.3)	242 (3.6)	962 (4.5)	
Low physical activity score, n (%)	6,952 (25.0)	1,480 (21.8)	5,472 (26.0)	<0.0001
Dietary fiber, g/1,000 kcal (IQR)	8.8 (7.2-10.7)	8.8 (7.3-10.7)	8.8 (7.2-10.7)	0.30
Fruit and vegetables, g/1,000 kcal (IQR)	161 (110-226)	162 (111-229)	161 (110-226)	0.22
SSB, g/1,000 kcal (IQR)	2.7 (0-42)	1.9 (0.0-37.0)	3.3 (0.0-44.1)	0.0007
Coffee, g/1,000 kcal (IQR)	204 (117-323)	211 (121-340)	202 (115-318)	<0.0001
Red/processed meat, g/1,000 kcal (IQR)	50.9 (27.6-66.1)	47.6 (33.9-62.1)	52.1 (38.7-67.3)	<0.0001
PNPLA3 rs738409 GG-genotype, n (%)	1,319 (4.5)	288 (4.4)	916 (4.5)	0.81
Top decile of PRS-MASLD, n (%)	2,696 (10)	627 (9.5)	2,069 (10.1)	0.72
Top decile of PRS-cirrhosis, n (%)	2,692 (10)	594 (9.0)	2,098 (10.3)	0.012
Top decile of PRS-cALT, n (%)	2,693 (10)	627 (9.5)	2,066 (10.1)	0.16
Number of participants, n (%)	4,549	1,157 (25.4)	3,392 (74.6)	—
Fasting plasma glucose, mmol/L (IQR)	5.4 (5.1-5.8)	5.2 (5.0-5.6)	5.4 (5.1-5.8)	<0.0001
HbA1c, % (IQR)	4.8 (4.5-5.0)	4.7 (4.5-5.0)	4.8 (4.5-5.1)	0.0005
HOMA-IR (IQR)	1.3 (0.9-1.9)	1.0 (0.6-1.4)	1.5 (1.0-2.1)	<0.0001
LDL, mmol/L (IQR)	4.1 (3.5-4.8)	3.9 (3.3-4.6)	4.2 (3.5-4.8)	<0.0001
HDL, mmol/L (IQR)	1.3 (1.1-1.6)	1.5 (1.2-1.7)	1.3 (1.1-1.6)	<0.0001
Triglycerides, mmol/L (IQR)	1.1 (0.9-1.5)	1.0 (0.8-1.3)	1.2 (0.9-1.6)	<0.0001
hsCRP, mg/L (IQR)	1.3 (0.6-2.6)	0.9 (0.5-1.8)	1.5 (0.7-2.9)	<0.0001

cALT, chronically elevated ALT; CLD, chronic liver disease; HOMA-IR, homeostatic model assessment for insulin resistance; MASLD, metabolic dysfunction-associated steatotic liver disease; MDCS, Malmö Diet and Cancer Study; MDCS-CC -Malmö Diet and Cancer Study, Cardiovascular Cohort; PRS, polygenic risk score; SSB, sugar-sweetened beverage.
**p* values for differences by metabolic health status from chi-square test for categorical variables (expressed as n (%)) and t-test for normally distributed continuous variables (expressed as mean (SD)) or Kruskal-Wallis test for continuous variables with skewed distribution (expressed as median (IQR)).

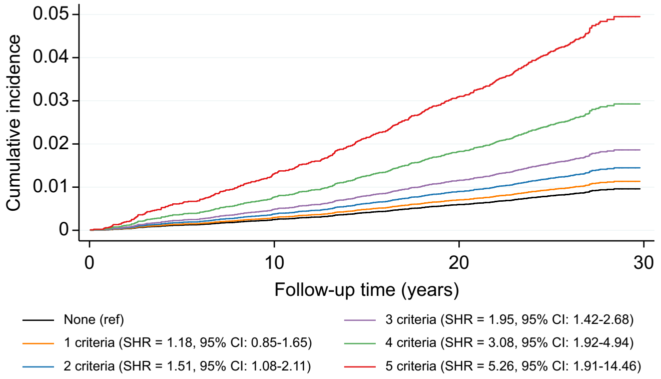

Fig. 2. Cumulative incidence of CLD by a number of adult cardiometabolic criteria for MASLD in the MDCS. The cumulative incidence and the SHR with 95% CIs were estimated using a competing risk regression model accounting for non-CLD deaths as competing events and adjusting for age and sex. CLD, chronic liver disease; MASLD, metabolic dysfunction-associated steatotic liver disease; MDCS, Malmö Diet and Cancer Study; SHR, subdistribution hazard ratio.

adjustment for age- and sex as well as after adjustment for prevalent diabetes mellitus, BMI, hypertension, and use of lipid-lowering drugs (Table S3). In the fully adjusted model, prevalent diabetes mellitus (HR 2.22; 95% CI 1.56-3.15; p = 8.3 x 10^{-6}), BMI (HR per SD increase = 1.26; 95% CI 1.13-1.40; p = 2.6 x 10^{-5}), waist circumference (HR per SD increase = 1.93; 95% CI 1.49-2.50; p = 6.3 x 10^{-7}) and HOMA-IR (HR per SD increase = 2.11; 95% CI 1.62-2.75; p = 2.8 x 10^{-8}) were associated with increased risk of CLD.

Lifestyle risk factors

The cumulative incidence of CLD was higher among those with an unhealthy compared to healthy lifestyle (Fig. 3). After adjustment for age, sex, and educational level, an unhealthy compared to a healthy lifestyle was associated with increased

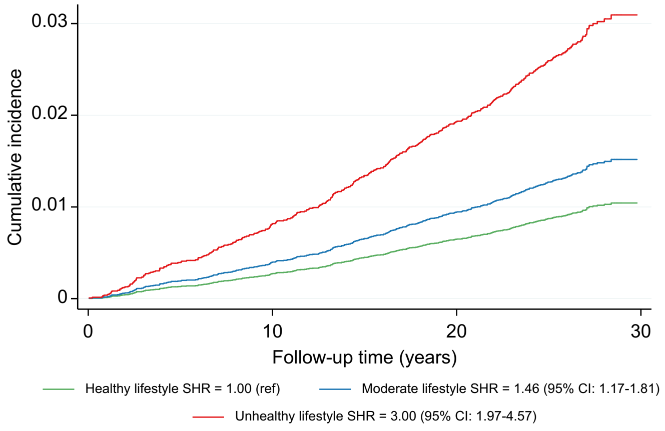

Fig. 3. Lifestyle risk score in relation to incident CLD in the MDCS (N = 27,737). The plot displays the cumulative incidence function by categories of lifestyle risk score based on presence of high alcohol intake, current smoking, physical inactivity and unhealthy diet (healthy lifestyle=zero risk factors; moderate lifestyle=1-2 risk factors; unhealthy lifestyle=3-4 risk factors). SHR and 95% CIs were estimated using a competing risk regression model accounting for non-CLD deaths as competing events and adjusting for age, sex and educational level. CLD, chronic liver disease; MDCS, Malmö Diet and Cancer Study; SHR, subdistribution hazard ratio.

risk of CLD (HR 3.97; 95% CI 2.59-6.10; p for trend across categories = 4.5 x 10^{-10}). Additional adjustment for prevalent diabetes mellitus, BMI, waist circumference, hypertension and use of lipid-lowering drugs had a very modest attenuating effect on the observed risk estimate (HR 3.72; 95% CI 2.41-5.72; p for trend across categories = 7.2 x 10^{-9}; data not tabulated). The risk estimates associated with an unhealthy lifestyle were stronger for SLD (HR 5.65; 95% CI 2.41-13.2; p = 6.7x10^{-5}) and cirrhosis (HR 4.75; 95% CI 2.61-8.67; p = 1.0 x 10^{-7}), but not HCC (HR 3.13; 95% CI 1.29-7.56; p = 0.011) (data not tabulated). All examined lifestyle risk factors were associated with a higher risk of CLD after mutual adjustment including current smoking (HR 1.71; 95% CI 1.33-2.19), high alcohol intake (HR 2.30; 95% CI 1.61-3.28), physical inactivity (HR 1.41; 95% CI 1.06-1.88), and an unhealthy diet (HR 1.53; 95% CI 1.02-2.30) (Table 2). Among the dietary components included in the diet risk score, we observed a protective effect of high fiber intake (HR per SD increase = 0.80; 95% CI 0.68-0.93) and coffee intake (HR per SD increase = 0.90; 95% CI 0.81-0.99). There was a non-significant tendency for a higher risk of CLD with higher intake of red and processed meats (HR 1.12; 95% CI 0.99-1.26; p = 0.061) (Table 2).

Genetic risk factors

Overall, risk of CLD increased linearly by increasing level of PRS (Fig. 4). Compared to the lowest deciles of polygenic risk, participants in the top deciles of PRS-MASLD (HR 2.98; 95% CI 1.08-8.19; p = 0.035), PRS-cirrhosis (HR 5.05; 95% CI 1.73-14.79; p = 0.0031), and PRS-cALT (HR 2.61; 95% CI 1.02-6.68; p = 0.045) had a higher risk of CLD (data not tabulated). The effect of individual genetic variants on CLD is shown in Table S5. Analyses confirmed an association between genetic variation in *PNPLA3, TM6SF2, SERPINA1,* and *ARHGEF28* with risk of CLD. Other genetic variants showed non-significant effects but, with a few exceptions, associations were directionally concordant with previously reported effects. The *PNPLA3* rs738409, PRS-MASLD, PRS-cirrhosis, and PRS-cALT were associated with all specific liver outcomes including SLD, cirrhosis and HCC. There was no significant heterogeneity in associations by age and sex but associations between PRS and liver outcomes tended to be stronger among those below age 60 years at baseline (Table S6).

Combined impact of cardiometabolic, lifestyle and genetic risk factors

Compared to a model including only age, sex and educational level (C-statistic = 0.6636), adding information on cardiometabolic, lifestyle and genetic risk factors all improved prediction (Table 3). In a model including age, sex, educational level, metabolic and lifestyle risk factors the C-statistic increased to 0.7211. Additional inclusion of the *PNPLA3* rs738409 genetic variant and the different PRSs significantly improved the C-statistic, however, the absolute increases in C-statistic were very modest (Table 3). On top of cardiometabolic and lifestyle risk factors, only the PRS-cirrhosis improved the C-statistic (C-statistic = 0.7345) above that of the *PNPLA3* rs738409 genetic variant (C-statistic = 0.7301). Overall, we found little evidence for heterogeneity in associations between lifestyle risk factors and risk of CLD depending on cardiometabolic risk factors. For the association between coffee

Table 2. Lifestyle risk factors for CLD in the MDCS (N = 27,991) and heterogeneity by number of cardiometabolic criteria at baseline.

Risk factor	n (cases)	HR (95% CI) Model 1*	*p* value	HR (95% CI) Model 2**	*p* value	$p_{interaction}$†
Educational level						0.90 (0.62)
Elementary or lower	11,731 (190)	1.00 (ref)	ref	1.00 (ref)	ref	
Middle school	7,299 (87)	0.79 (0.61-1.03)	0.079	0.82 (0.63-1.06)	0.13	
High school	4,924 (75)	0.87 (0.66-1.13)	0.29	0.90 (0.69-1.19)	0.47	
University degree	3,967 (28)	0.43 (0.29-0.64)	3.9×10^{-5}	0.47 (0.31-0.71)	2.7×10^{-4}	
Smoking status						0.014 (0.58)
Never	10,612 (120)	1.00 (ref)	ref	1.00 (ref)		
Former	9,461 (115)	0.96 (0.74-1.25)	0.77	0.92 (0.70-1.19)	0.52	
Current	7,906 (146)	1.90 (1.49-2.43)	3.2×10^{-7}	1.71 (1.33-2.19)	3.1×10^{-5}	
Alcohol consumption						0.97 (0.80)
Zero	1,699 (27)	1.59 (1.06-2.36)	0.024	1.41 (0.93-2.14)	0.10	
Low	20,308 (243)	1.00 (ref)	ref	1.00 (ref)	ref	
Moderate	4,780 (73)	1.14 (0.87-1.49)	0.33	1.15 (0.88-1.50)	0.32	
High	1,204 (39)	2.43 (1.72-3.43)	4.7×10^{-7}	2.30 (1.61-3.28)	4.3×10^{-6}	
Physical activity score						0.50 (0.35)
Quartile 4	6,947 (83)	1.00 (ref)	ref	1.00 (ref)	ref	
Quartile 3	6,937 (99)	1.18 (0.88-1.58)	0.27	1.19 (0.88-1.59)	0.25	
Quartile 2	6,969 (75)	0.90 (0.66-1.24)	0.53	0.89 (0.65-1.22)	0.46	
Quartile 1	6,952 (121)	1.55 (1.17-2.05)	2.3×10^{-3}	1.41 (1.06-1.88)	0.017	
Diet risk score						0.53 (0.36)
Healthy (13-15 points)	4,548 (39)	1.00 (ref)	ref	1.00 (ref)	ref	
Moderate (8-12 points)	19,778 (263)	1.34 (0.95-1.89)	0.092	1.18 (0.83-1.68)	0.34	
Unhealthy (5-7 points)	3,665 (80)	1.96 (1.31-2.91)	9.3×10^{-4}	1.53 (1.02-2.30)	0.041	
Dietary fiber, per SD increase	27,991 (382)	0.73 (0.66-0.81)	1.2×10^{-9}	0.80 (0.68-0.93)	3.7×10^{-3}	0.11 (0.16)
Fruit/vegetables, per SD increase	27,991 (382)	0.82 (0.74-0.90)	6.9×10^{-5}	1.06 (0.91-1.24)	0.43	0.88 (0.72)
SSB, per SD increase	27,991 (382)	1.01 (0.91-1.12)	0.84	0.97 (0.88-1.08)	0.58	0.42 (0.58)
Coffee, per SD increase	27,991 (382)	0.94 (0.86-1.04)	0.24	0.90 (0.81-0.99)	0.029	0.024 (0.040)
Red/processed meat, per SD increase	27,991 (382)	1.19 (1.06-1.33)	3.6×10^{-3}	1.12 (0.99-1.26)	0.061	0.96 (0.38)

CLD, chronic liver disease; HR, hazard ratio; MDCS, Malmö Diet and Cancer Study; SD, standard deviation; SSB, sugar-sweetened beverage.
HRs and 95% CIs were estimated using Cox proportional hazards models.
*Model 1 adjusted for age and sex.
**Model 2 adjusted for age and sex further included all covariates in the table except for individual dietary components to assess mutually independent effects of lifestyle risk factors. For individual dietary components, model 2 was adjusted for all covariates in the table except diet risk score.
†*p* value for multiplicative interaction between individual risk factors in the table with number of fulfilled cardiometabolic criteria at baseline adjusting for age and sex, and *p* value for interaction with mutual adjustment for all included covariates as outlined for Model 2 in parenthesis.

intake and CLD, however, there was some evidence suggesting that the protective effect was limited to those with pre-existing cardiometabolic risk factors (*p* interaction = 0.040; Table 2). Similarly, we observed limited evidence for heterogeneity in effects of cardiometabolic and lifestyle risk factors on CLD based on underlying genetic predisposition (Table S5).

Discussion

This large prospective study confirms the importance of cardiometabolic dysfunction in risk of CLD and importantly provides novel evidence suggesting that lifestyle risk factors increase risk of CLD independently of underlying cardiometabolic health and genetic predisposition. While our results suggest that novel PRSs for MASLD and cirrhosis are strongly associated with higher risk of CLD, they appear to have limited utility for prediction of CLD on top of cardiometabolic and lifestyle risk factors in the general population.

We confirmed previously known associations between several cardiometabolic risk factors and risk of CLD in the general population. Diabetes mellitus was associated with a more than two-fold increased risk of CLD, which is in line with previously reported risk estimates.[21,22] There appear to be complex bidirectional pathways between MASLD, type 2 diabetes and obesity. A Mendelian randomization study, using genetic instruments to avoid issues of confounding and reverse causation, suggested that while genetically driven type 2 diabetes, obesity and central obesity increase the risk of MASLD, MASLD also promotes development of type 2 diabetes and central obesity.[23] In line with previous studies,[24] we also showed that, compared to BMI, waist circumference was a stronger predictor of CLD. A recent Mendelian randomization analysis suggested that waist circumference causally increases the risk of MASLD after adjusting for BMI, while BMI was not associated with MASLD after adjusting for waist circumference.[25] Among the other cardiometabolic traits examined in our study only HOMA-IR showed a significant and independent association with increased risk of CLD. We found no support for an association between dyslipidemia or the use of lipid-lowering drugs with CLD, which is concordant with a recent Mendelian randomization analysis that did not support dyslipidemia as a causal risk factor for MASLD.[26] Arterial hypertension has previously been linked to risk of severe liver-related outcomes.[27–30] Recent studies suggested that both measured and genetically elevated blood pressure increase the risk of liver disease.[31,32] While we observed that hypertension was associated with a higher risk of CLD, the association was not significant after adjustment for other cardiometabolic risk factors including diabetes and adiposity.

Low educational level or socioeconomic status has previously been linked to increased risk of MASLD as well as HCC,[33–36] a finding that was confirmed in our study. Most importantly, we found that an overall unhealthy lifestyle was associated with a three- to four-fold increased risk of CLD

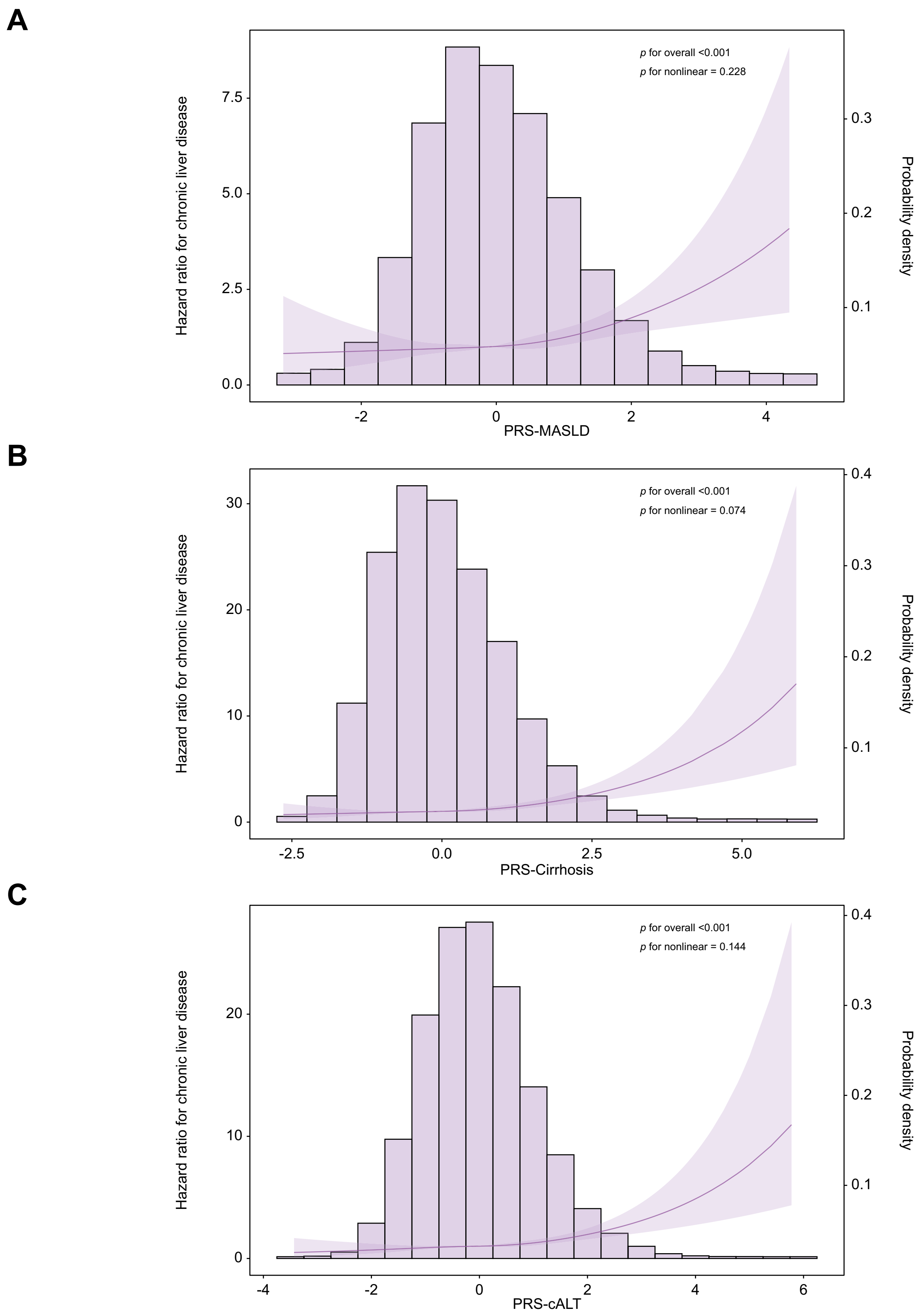

Fig. 4. PRS and incident CLD in the MDCS (N = 26,965). (A-C) The distribution (histograms) of participants across the different PRSs and their association with incident CLD using a Cox regression model adjusting for age and sex with a fitted restricted cubic spline. The solid line represents the HR and the shaded area the 95% CI. The plots show the *p* values for the overall (linear effect) and for nonlinearity. cALT, chronically elevated ALT; CLD, chronic liver disease; MASLD, metabolic dysfunction-associated steatotic liver disease; MDCS, Malmö Diet and Cancer Study; PRS, polygenic risk score.

Table 3. Harrell's C (concordance) statistic for the added benefit of metabolic, lifestyle and genetic risk factors in prediction of CLD in the MDCS (N = 26,725 with complete data on all included predictors).

Model	C-statistic	*p* value (LR)
Age, sex, and educational level	0.6636	–
+ Metabolic risk factors*	0.6968	<0.00001
+ Lifestyle risk factors**	0.7017	<0.00001
+ *PNPLA3* rs738409	0.6766	0.0001
+ PRS-MASLD	0.6739	<0.00001
+ PRS-cALT	0.6816	<0.00001
+ PRS-cirrhosis	0.6830	<0.00001
Age, sex, education, metabolic and lifestyle risk factors	0.7211	–
+ *PNPLA3* rs738409	0.7301	0.0002
+ PRS-MASLD	0.7269	0.0001
+ PRS-cALT	0.7296	<0.00001
+ PRS-cirrhosis	0.7345	<0.00001

cALT, chronically elevated ALT; CLD, chronic liver disease; LR, likelihood ratio; MASLD, metabolic dysfunction-associated steatotic liver disease; MDCS, Malmö Diet and Cancer Study; PRS, polygenic risk score.
Models were compared using the LR test.
*Metabolic risk factors included prevalent diabetes mellitus, BMI, waist circumference, hypertension and use of lipid-lowering medications.
**Lifestyle risk factors included smoking status, alcohol consumption, physical activity, and diet risk score.

independently of educational level and cardiometabolic dysfunction. In our cohort, approximately 4% were classified as high alcohol consumers (>40 g/day for men and >30 g/day for women), and high compared to low alcohol intake was associated with a 78% increased risk of CLD. Underreporting of alcohol intake may however bias this association towards the null. A recent review concluded that smoking is associated with both development and progression of liver disease.[37] While the observed increased risk associated with smoking in our study is significant on the population level it may hold less impact on the individual level and advice on smoking cessation should continue to emphasize other important health benefits.[37] Participants in the upper three quartiles of physical activity compared to the lowest quartile had a lower risk of CLD, suggesting a potential threshold level above which no additional benefit is observed. The absence of a clear dose-response association may also reflect the use of self-reported physical activity. In a study from the UK Biobank using accelerometer-derived physical activity level, participants with high physical activity had a dose-dependent lower risk of overall CLD as well as MASLD.[38]

Studies examining the role of diet in CLD compared to MASLD are generally scarce and hampered by small sample sizes, with substantial heterogeneity between studies. A meta-analysis of the role of dietary patterns in MASLD suggested that Western dietary patterns (typically high in red/processed meat and refined grains) increased risk of MASLD while more health-conscious food patterns (*e.g.*, Mediterranean-type dietary patterns) decreased risk of MASLD.[39] Similar findings were observed in the UK Biobank, suggesting that high consumption of red meat and lower consumption of fruits, cereals, and dietary fiber are associated with higher risk of MASLD, cirrhosis and HCC.[40] We found suggestive protective effects of dietary fiber and coffee intake while higher intake of red and processed meat was associated with higher risk of CLD in our cohort. In an umbrella review, a benefit of coffee consumption on liver fibrosis was seen among patients with established MASLD, but no effect on the incidence of MASLD was observed.[41] In the UK Biobank study, coffee drinkers had a lower risk of CLD and death from CLD, and a lower risk of HCC,[42] which is in line with observations from our cohort. High consumption of red and processed meat has been convincingly linked to type 2 diabetes in observational studies.[43] For the role of red and processed meat in liver disease, large prospective studies are generally lacking. Cross-sectional studies indicate an association between red and processed meats with MASLD and insulin resistance.[44] Additional large prospective studies are needed to fully elucidate the role of specific dietary components and overall dietary patterns on the progression of SLD and incidence of CLD to better inform evidence-based dietary guidelines or guide future dietary intervention studies.

In contrast to some previous investigations,[9,14] we found that the effects of cardiometabolic and lifestyle risk factors were largely independent of genetic predisposition as assessed by the *PNPLA3* genetic variant, the PRS-MASLD, the PRS-cirrhosis, and the PRS-cALT. However, these interaction analyses may be underpowered in our study population and are not fully comparable to previous studies due to the use of a composite CLD outcome. Interestingly, compared to the *PNPLA3* genetic variant, only the PRS-cirrhosis model added additional information for prediction of CLD in our cohort. There is an overlap in genetic variants associated with a wide range of liver-related outcomes including SLD, cirrhosis and HCC. Emdin *et al.*[9] utilized this for their multi-trait GWAS on cirrhosis, whereas previous genetic investigations have tried to examine each etiological subtype of SLD or cirrhosis separately resulting in small sample sizes. Since pathological processes may, to a large extent, be shared regardless of underlying etiology, future GWAS could harness the potentially improved statistical power in combining several related liver outcomes in order to better identify genetic variants that predict more severe liver disease. Although all three PRSs were strongly associated with a higher risk of CLD, the added benefit for prediction was marginal.

This finding is in line with a previous investigation suggesting no benefit of adding PRSs to established risk scores, including routine biomarkers such as the aspartate aminotransferase-to-platelet ratio index and the fibrosis-4 index.[45] A plausible explanation for the lack of benefit of including the PRSs on top of cardiometabolic and lifestyle risk factors in our study could be the pleiotropic nature of most of the included genetic variants. Several of the so far identified genetic variants for MASLD and cirrhosis are known to also affect several cardiometabolic traits.

The main strengths of this study include the use of a large population-based study with extensive data collection, including dietary assessment of high relative validity, direct measurements of anthropometrics and blood pressure, and GWAS genotyping. Further, the unique personal identification number held by all inhabitants in Sweden allows for registry linkage to assess outcomes and thereby low loss to follow-up (<0.5%, due to emigration from Sweden). The included diagnoses in the primary endpoint are likely to lead to hospitalization or death and are therefore well captured by the national patient registries in Sweden which are both validated and provide outcome data of high quality. Our findings have several important implications. First, our data suggest an important role of lifestyle risk factors for the development of CLD that is largely independent of underlying cardiometabolic risk factors and genetic predisposition. This finding has importance for the primary prevention of CLD, suggesting that public health strategies in line with current recommendations for prevention of cardiometabolic diseases will

likely lower the incidence of CLD. Secondly, while great efforts have been made in recent years to highlight the polygenic nature of lifestyle-related liver disease, so far adding genetic information to better predict incident CLD has limited utility on top of established risk factors.

While overall sample size was large and follow-up time considerable, the number of CLD events was limited, which reduces statistical power to detect weak effects as well as potential multiplicative interactions. The observational nature of the study hampers causal inference and residual confounding by covariates adjusted for (due to measurement error) and unmeasured confounders may impact observed associations. This is particularly important for assessment of the role of lifestyle risk factors which are self-reported and thus subjected to both measurement error and bias. We examined the association between a select number of dietary components separately and combined this into a diet risk score. Since not all included components were associated with CLD, the overall impact of an unhealthy diet as defined in our study may thus be underestimated. In observational settings a healthy diet can be defined using different approaches. In our study we opted to focus on a smaller set of dietary factors previously known to be associated with both liver disease and cardiometabolic diseases. The use of unweighted diet and lifestyle risk scores (*i.e.* assigning equal weight to all components) will also attenuate observed associations towards the null since risk factors that confer a very high risk (*i.e.* alcohol intake) will be counted as equal to risk factors conferring a more modest effect (*i.e.* diet). However, the approach used still allows for a sufficient ranking of individuals and assigning relative weights is problematic since there is a lack of consensus regarding the proposed relative effects of the included risk factors. Unfortunately, we had no possibility to ascertain presence of hepatic steatosis or other more severe liver damage at baseline apart from pre-existing diagnoses of CLD or other liver diseases. It is however plausible that the observed associations among those with poor cardiometabolic health would be similar in a population with verified MASLD due to the expected high prevalence of steatosis in this subpopulation. A major limitation is that we also lacked data on liver enzymes and liver function and were therefore not able to examine if the examined predictors for CLD in our study add predictive value above that of established risk scores. Notably, the C-indexes of established scores typically exceed those reported in our study, which further highlights the importance of such established markers for prediction of CLD in a general population.[45]

In this comprehensive analysis of risk factors for CLD in a general population, we confirm the importance of cardiometabolic perturbations. We further validate the association between several well-known and more novel genetic variants, which have been associated with MASLD and cirrhosis, in relation to the incidence of CLD in the general population. High polygenic risk of MASLD and cirrhosis conferred an increased risk of CLD but had limited predictive capability on top of cardiometabolic and lifestyle risk factors. A healthy lifestyle that promotes cardiometabolic health is likely to be beneficial for lowering the risk of CLD in the general population, irrespective of pre-existing cardiometabolic dysfunction and genetic predisposition. Since the absolute risk of CLD is low compared to that of cardiometabolic diseases it is reassuring that the same targets for primary prevention are highly relevant for CLD.

Affiliations

[1]*Department of Clinical Sciences in Malmö, Lund University, Malmö, Sweden;* [2]*Skåne University Hospital, Malmö, Sweden;* [3]*Gastroenterology Research Unit, Department of Clinical Sciences in Malmö, Lund University, Malmö, Sweden;* [4]*Department of Surgery and Gastroenterology, Skåne University Hospital, Malmö, Sweden*

Abbreviations

ALD, alcohol-related liver disease; cALT, chronically elevated ALT; CLD, chronic liver disease; HOMA-IR, homeostatic model assessment for insulin resistance; HR, hazard ratio; MASLD, metabolic dysfunction-associated steatotic liver disease; MDCS, Malmö Diet and Cancer Study; MDCS-CC, Malmö Diet and Cancer Study, Cardiovascular Cohort; PRS, polygenic risk score; SD, standard deviation; SLD, steatotic liver disease

Financial support

ID was supported by grants from the Swedish Society for Medical Research, Dr P Håkansson foundation, and the Påhlsson Foundation. The study was additionally supported by grants to MO-M from the Swedish Research Council (2021-03291), the Swedish Heart and Lung Foundation (20200711), the regional Region Skåne County Council ALF grant (2022-0258) and the Novo Nordisk Foundation (NNF20OC0063886). The funders had no role in conceptualization, design, data collection, analysis, decision to publish or preparation of the manuscript.

Conflict of interest

The authors declare that they have no conflict of interest.

Please refer to the accompanying ICMJE disclosure forms for further details.

Authors' contributions

Study Concept and Design: ID. Data Analysis: ID. Manuscript Preparation: ID. Critical Manuscript Review and interpretation of results: All authors.

Data availability statement

Datasets analyzed during the current study are not publicly available due to the nature of the sensitive personal data and study materials. However, procedures for sharing data, analytic methods, and study materials for reproducing the results following Swedish legislation can be arranged by contacting the corresponding author or study organization (https://www.malmo-kohorter.lu.se/malmo-kost-cancer-mkc).

Ethics approval and consent to participate

The study was conducted in ethical accordance with the World Medical Association Declaration of Helsinki. The protocol was approved by the Regional Ethical Review Board in Lund, Sweden (Dnr § LU 51-90, 2007/166). Written and oral informed consent for inclusion and publication was given by all subjects prior to participation.

Acknowledgements

We acknowledge support from Lund University Infrastructure grant "Malmö population-based cohorts" (STYR 2019/2046), Swedish Foundation for Strategic Research (IRC LUDC), and Swedish Research Council (SFO-EXODIAB). We acknowledge the Regeneron Genetics Center (RGC) for providing genotype data for the MDCS cohort, RGC contributors listed below.

RGC Management & Leadership Team: Gonçalo Abecasis, D. Phil.[1], Adolfo Ferrando, M.D., Ph.D.[1], Aris Baras, M.D.[1], Michael Cantor, M.D.[1], Giovanni Coppola, M.D.[1], Andrew Deubler, M.P.S.[1], Aris Economides, Ph.D.[1], Luca A Lotta, M.D., Ph.D.[1], John D Overton, Ph.D.[1], Jeffrey G Reid, Ph.D.[1], Alan Shuldiner, M.D.[1], Katherine Siminovitch, M.D.[1] Contribution: All authors contributed to securing funding, study design and oversight. All authors reviewed the final version of the manuscript. Sequencing & Lab Operations: John D Overton, Ph.D.[1], Christina Beechert[1], Erin D Brian[1], Laura M Cremona, Ph.D.[1], Hang Du1, Caitlin Forsythe, M.S.[1], Zhenhua Gu, M.S.[1], Kristy Guevara, M.S.[1], Michael Lattari[1], Alexander Lopez, M.S.[1], Kia Manoochehri[1], Prathyusha Challa, M.S.[1], Manasi Pradhan, M.S.[1], Raymond Reynoso[1], Ricardo Schiavo[1], Maria Sotiropoulos Padilla, M.S.[1], Chenggu Wang, M.S.[1], Sarah E Wolf, M.S.[1]

Contribution: Performed and are responsible for sample genotyping and exome sequencing, conceived and are responsible for laboratory automation, and responsible for sample tracking and the library information management system.

Clinical Informatics: Michael Cantor M.D. [1], Amelia Averitt, Ph.D.[1], Nilanjana Banerjee, Ph.D.[1], Dadong Li, Ph.D.[1], Sameer Malhotra, M.D. [1], Justin Mower, Ph.D.[1], Mudasar Sarwar, Deepika Sharma, Ph.D.[1], Jeffrey C Staples, Ph.D.[1], Sean Yu, Ph.D.[1], Aaron Zhang, Ph.D.[1] Contribution: Development and validation of clinical phenotypes used to identify study participants and (when applicable) controls. Genome Informatics & Data Engineering: Jeffrey G Reid, Ph.D.[1], Mona Nafde, M.S.[1], George Mitra[1], Sujit Gokhale[1], Andrew Bunyea[1], Krishna Pawan Punuru, M.S.[1], Sanjay Sreeram[1], Gisu Eom[1], Sujit Gokhale[1], Benjamin Sultan, M.S.[1], Rouel Lanche[1], Vrushali Mahajan[1], Eliot Austin[1], Sean O'Keeffe, Ph.D.[1], Razvan Panea, Ph.D.[1], Tommy Polanco[1], Ayesha Rasool, M.S.[1], William Salerno, Ph.D.[1], Xiaodong Bai, Ph.D.[1], Lance Zhang, M.S.[1], Boris Boutkov, Ph.D.[1], Evan Edelstein[1], Alexander Gorovits, Ph.D.[1], Ju Guan, Ph.D.[1], Lukas Habegger, Ph.D.[1], Alicia Hawes[1], Olga Krasheninina, M.S.[1], Samantha Zarate, Ph.D.[1], Adam J Mansfield[1], Evan K Maxwell, Ph.D.[1], Suganthi Balasubramanian, Ph.D.[1], Suying Bao, Ph.D.[1], Kathie Sun, Ph.D.[1], Chuanyi Zhang, Ph.D.[1]

Contribution: Performed and are responsible for analysis needed to produce exome and genotype data, provided compute infrastructure development and operational support, provided variant and gene annotations and their functional interpretation of variants, and conceived and are responsible for creating, developing, and deploying analysis platforms and computational methods for analyzing genomic data.

Analytical Genetics and Data Science: Gonçalo Abecasis, D. Phil.[1], Manuel Allen Revez Ferreira, Ph.D.[1], Joshua Backman, Ph.D.[1], Kathy Burch, Ph.D.[1], Adrian Campos, Ph.D.[1], Lei Chen, Ph.D.[1], Sam Choi, Ph.D.[1], Amy Damask, Ph.D.[1], Liron Ganel, Ph.D.[1], Sheila Gaynor, Ph.D.[1], Benjamin Geraghty, Ph.D.[1], Arkopravo Ghosh, M.S.[1], Salvador Romero Martinez[1], Christopher Gillies, Ph.D.[1], Lauren Gurski[1], Joseph Herman, D. Phil.[1], Eric Jorgenson, Ph.D.[1], Tyler Joseph, Ph.D.[1], Michael Kessler, Ph.D.[1], Jack Kosmicki, Ph.D.[1], Nan Lin, Ph.D.[1], Adam Locke, Ph.D.[1], Priyanka Nakka, Ph.D.[1], Jonathan Marchini, Ph.D.[1], Karl Landheer, Ph.D.[1], Olivier Delaneau, Ph.D.[1], Maya Ghoussaini, Ph.D.[1], Anthony Marcketta, M.S.[1], Joelle Mbatchou, Ph.D.[1], Arden Moscati, Ph.D.[1], Aditeya Pandey, Ph.D.[1], Anita Pandit, M.S.[1], Charles Paulding, Ph.D.[1], Jonathan Ross[1], Carlo Sidore, Ph.D.[1], Eli Stahl, Ph.D.[1], Maria Suciu, Ph.D.[1], Timothy Thornton, Ph.D.[1], Peter VandeHaar, M.S.1, Sailaja Vedantam, Ph.D.[1], Scott Vrieze, Ph.D.[1], Jingning Zhang, Ph.D.[1], Rujin Wang, Ph.D.[1], Kuan-Han Wu, Ph.D.[1], Bin Ye, Ph.D.[1], Blair Zhang, Ph.D.[1], Andrey Ziyatdinov, Ph.D.[1], Yuxin Zou, Ph.D.[1], Olivier Delaneau, Ph.D.[1], Maya Ghoussaini, Ph.D.[1], Jingning Zhang, Ph.D.[1], Kyoko Watanabe, Ph.D.[1], Mira Tang.

Contribution: Development of statistical analysis plans. QC of genotype and phenotype files and generation of analysis ready datasets. Development of statistical genetics pipelines and tools and use thereof in generation of the association results. QC, review and interpretation of results. Generation and formatting of results for manuscript figures.

Therapeutic Area Genetics: Adolfo Ferrando, M.D., Ph.D.[1], Giovanni Coppola, M.D.[1], Luca A Lotta, M.D., Ph.D.[1], Alan Shuldiner, M.D.[1], Katherine Siminovitch, M.D.[1], Brian Hobbs, M.D.[1], Jon Silver, Ph.D.[1], William Palmer, Ph.D.[1], Rita Guerreiro, Ph.D.[1], Amit Joshi, Ph.D.[1], Antoine Baldassari, Ph.D.[1], Cristen Willer, D. Phil.[1], Sarah Graham, Ph.D.[1], Ernst Mayerhofer, M.D.[1], Mary Haas, Ph.D.[1], Niek Verweij, Ph.D.[1], George Hindy, Ph.D.[1], Jonas Bovijn, M.D.[1], Tanima De, Ph.D.[1], Parsa Akbari, Ph.D.[1], Luanluan Sun, Ph.D.[1], Olukayode Sosina, Ph.D.[1], Arthur Gilly, Ph.D.[1], Peter Dornbos, Ph.D.[1], Juan Rodriguez-Flores, Ph.D.[1], Moeen Riaz, Ph.D.[1], Manav Kapoor, Ph.D.[1], Gannie Tzoneva, Ph.D.[1], Momodou W Jallow, Ph.D.[1], Anna Alkelai, Ph.D.[1], Giovanni Coppola, M.D.[1], Ariane Ayer[1], Veera Rajagopal, M.D.[1], Sahar Gelfman, Ph.D.[1], Vijay Kumar, Ph.D.[1], Jacqueline Otto, Ph.D.[1], Neelroop Parikshak, M.D.[1], Aysegul Guvenek, Ph.D.[1], Jose Bras, Ph.D.[1], Silvia Alvarez, Ph.D.[1], Jessie Brown, Ph.D.[1], Jing He, Ph.D.[1], Hossein Khiabanian, Ph.D.[1]., Joana Revez, Kimberly Skead, Valentina Zavala.

Contribution: Development of study design and analysis plans. Development and QC of phenotype definitions. QC, review, and interpretation of association results.

Research Program Management & Strategic Initiatives: Lyndon J Mitnaul, Ph.D.[1], Marcus B Jones, Ph.D.[1], Esteban Chen, M.S.[1], Michelle G LeBlanc, Ph.D.[1], Jason Mighty, Ph.D.[1], Nirupama Nishtala, Ph.D.[1], Nadia Rana, Ph.D.[1], Jaimee Hernandez, M.S.[1], Jennifer Rico-Varela, Ph.D.[1]

Contribution: Contributed to the management and coordination of all research activities, planning and execution, managed the review of the project. Senior Partnerships & Business Operations: Alison Fenney, Ph.D., MBA[1], Randi Schwartz, MBA[1], Jody Hankins, Ph.D., MBA[1], Samuel Hart, J.D.[1] Contribution: Contributed to the management, planning, execution, and negotiation of new and existing agreements.

Business Operations & Administrative Coordinators: Ann Perez-Beals[1], Gina Solari[1], Jaimee Hernandez, M.S.[1], Johannie Rivera-Picart[1], Michelle Pagan[1], Sunilbe Siceron[1].

Contribution: coordinate all administrative activities with internal stakeholders and external collaborators.

Affiliations:

1. Regeneron Genetics Center, Tarrytown, NY, USA.

Supplementary data

Supplementary data to this article can be found online at https://doi.org/10.1016/j.jhepr.2024.101105.

References

Author names in bold designate shared co-first authorship

[1] Devarbhavi H, Asrani SK, Arab JP, et al. Global burden of liver disease: 2023 update. J Hepatol 2023;79(2):516–537.
[2] Pimpin L, Cortez-Pinto H, Negro F, et al. Burden of liver disease in Europe: epidemiology and analysis of risk factors to identify prevention policies. J Hepatol 2018;69(3):718–735.
[3] Rinella ME, Lazarus JV, Ratziu V, et al. A multi-society Delphi consensus statement on new fatty liver disease nomenclature. J Hepatol 2023;79(6):1542–1556.
[4] Riazi K, Azhari H, Charette JH, et al. The prevalence and incidence of NAFLD worldwide: a systematic review and meta-analysis. Lancet Gastroenterol Hepatol 2022;7(9):851–861.
[5] Amonker S, Houshmand A, Hinkson A, et al. Prevalence of alcohol-associated liver disease: a systematic review and meta-analysis. Hepatol Commun 2023;7(5).
[6] Idalsoaga F, Kulkarni AV, Mousa OY, et al. Non-alcoholic fatty liver disease and alcohol-related liver disease: two intertwined entities. Front Med (Lausanne) 2020;7:448.
[7] **Vujkovic M**, **Ramdas S**, Lorenz KM, et al. A multiancestry genome-wide association study of unexplained chronic ALT elevation as a proxy for nonalcoholic fatty liver disease with histological and radiological validation. Nat Genet 2022;54(6):761–771.
[8] **Chen Y**, **Du X**, Kuppa A, et al. Genome-wide association meta-analysis identifies 17 loci associated with nonalcoholic fatty liver disease. Nat Genet 2023;55(10):1640–1650.
[9] Emdin CA, Haas M, Ajmera V, et al. Association of genetic variation with cirrhosis: a multi-trait genome-wide association and gene-environment interaction study. Gastroenterology 2021;160(5):1620–1633 e13.
[10] Younossi ZM, Zelber-Sagi S, Henry L, et al. Lifestyle interventions in nonalcoholic fatty liver disease. Nat Rev Gastroenterol Hepatol 2023;20(11):708–722.
[11] Fernandez T, Vinuela M, Vidal C, et al. Lifestyle changes in patients with non-alcoholic fatty liver disease: a systematic review and meta-analysis. PLoS One 2022;17(2):e0263931.
[12] Nobili V, Carter-Kent C, Feldstein AE. The role of lifestyle changes in the management of chronic liver disease. BMC Med 2011;9:70.
[13] He P, Zhang Y, Ye Z, et al. A healthy lifestyle, Life's Essential 8 scores and new-onset severe NAFLD: a prospective analysis in UK Biobank. Metabolism 2023;146:155643.
[14] Luukkonen PK, Farkkila M, Jula A, et al. Abdominal obesity and alcohol use modify the impact of genetic risk for incident advanced liver disease in the general population. Liver Int 2023;43(5):1035–1045.
[15] Berglund G, Elmstahl S, Janzon L, et al. The malmo diet and cancer study. Design and feasibility. J Intern Med 1993;233(1):45–51.
[16] Wirfalt E, Mattisson I, Johansson U, et al. A methodological report from the Malmo Diet and Cancer study: development and evaluation of altered routines in dietary data processing. Nutr J 2002;1:3.
[17] Manjer J, Carlsson S, Elmstahl S, et al. The Malmo Diet and Cancer Study: representativity, cancer incidence and mortality in participants and non-participants. Eur J Cancer Prev 2001;10(6):489–499.
[18] Hedblad B, Nilsson P, Janzon L, et al. Relation between insulin resistance and carotid intima-media thickness and stenosis in non-diabetic subjects. Results from a cross-sectional study in Malmo, Sweden. Diabet Med 2000;17(4):299–307.
[19] Eslam M, Newsome PN, Sarin SK, et al. A new definition for metabolic dysfunction-associated fatty liver disease: an international expert consensus statement. J Hepatol 2020;73(1):202–209.

[20] Hindy G, Aragam KG, Ng K, et al. Genome-wide polygenic score, clinical risk factors, and long-term trajectories of coronary artery disease. Arterioscler Thromb Vasc Biol 2020;40(11):2738–2746.
[21] Adami HO, Chow WH, Nyren O, et al. Excess risk of primary liver cancer in patients with diabetes mellitus. J Natl Cancer Inst 1996;88(20):1472–1477.
[22] Pearson-Stuttard J, Papadimitriou N, Markozannes G, et al. Type 2 diabetes and cancer: an umbrella review of observational and mendelian randomization studies. Cancer Epidemiol Biomarkers Prev 2021;30(6):1218–1228.
[23] Liu Z, Zhang Y, Graham S, et al. Causal relationships between NAFLD, T2D and obesity have implications for disease subphenotyping. J Hepatol 2020;73(2):263–276.
[24] Andreasson A, Carlsson AC, Onnerhag K, et al. Waist/hip ratio better predicts development of severe liver disease within 20 Years than body mass index: a population-based cohort study. Clin Gastroenterol Hepatol 2017;15(8):1294–12301 e2.
[25] Gagnon E, Pelletier W, Gobeil E, et al. Mendelian randomization prioritizes abdominal adiposity as an independent causal factor for liver fat accumulation and cardiometabolic diseases. Commun Med (Lond) 2022;2:130.
[26] Li Z, Zhang B, Liu Q, et al. Genetic association of lipids and lipid-lowering drug target genes with non-alcoholic fatty liver disease. EBioMedicine 2023;90:104543.
[27] Singh S, Allen AM, Wang Z, et al. Fibrosis progression in nonalcoholic fatty liver vs nonalcoholic steatohepatitis: a systematic review and meta-analysis of paired-biopsy studies. Clin Gastroenterol Hepatol 2015;13(4):643–654. e1-9;quiz e39-40.
[28] Bjorkstrom K, Franzen S, Eliasson B, et al. Risk factors for severe liver disease in patients with type 2 diabetes. Clin Gastroenterol Hepatol 2019;17(13):2769–27675 e4.
[29] Kanwal F, Kramer JR, Li L, et al. Effect of metabolic traits on the risk of cirrhosis and hepatocellular cancer in nonalcoholic fatty liver disease. Hepatology 2020;71(3):808–819.
[30] Stocks T, Van Hemelrijck M, Manjer J, et al. Blood pressure and risk of cancer incidence and mortality in the Metabolic Syndrome and Cancer Project. Hypertension 2012;59(4):802–810.
[31] Aberg F, Kantojarvi K, Mannisto V, et al. Association between arterial hypertension and liver outcomes using polygenic risk scores: a population-based study. Sci Rep 2022;12(1):15581.
[32] Yuan S, Chen J, Li X, et al. Lifestyle and metabolic factors for nonalcoholic fatty liver disease: Mendelian randomization study. Eur J Epidemiol 2022;37(7):723–733.
[33] Vilar-Gomez E, Nephew LD, Vuppalanchi R, et al. High-quality diet, physical activity, and college education are associated with low risk of NAFLD among the US population. Hepatology 2022;75(6):1491–1506.
[34] Xie J, Huang H, Liu Z, et al. The associations between modifiable risk factors and nonalcoholic fatty liver disease: a comprehensive Mendelian randomization study. Hepatology 2023;77(3):949–964.
[35] Vaz J, Midlov P, Eilard MS, et al. Targeting population groups with heavier burden of hepatocellular carcinoma incidence: a nationwide descriptive epidemiological study in Sweden. Int J Cancer 2022; 151(2):229–239.
[36] Wang Y, Kong L, Ye C, et al. Causal impacts of educational attainment on chronic liver diseases and the mediating pathways: Mendelian randomization study. Liver Int 2023;43(11):2379–2392.
[37] Marti-Aguado D, Clemente-Sanchez A, Bataller R. Cigarette smoking and liver diseases. J Hepatol 2022;77(1):191–205.
[38] Schneider CV, Zandvakili I, Thaiss CA, et al. Physical activity is associated with reduced risk of liver disease in the prospective UK Biobank cohort. JHEP Rep 2021;3(3):100263.
[39] Hassani Zadeh S, Mansoori A, Hosseinzadeh M. Relationship between dietary patterns and non-alcoholic fatty liver disease: a systematic review and meta-analysis. J Gastroenterol Hepatol 2021;36(6):1470–1478.
[40] Guo W, Ge X, Lu J, et al. Diet and risk of non-alcoholic fatty liver disease, cirrhosis, and liver cancer: a large prospective cohort study in UK biobank. Nutrients 2022;14(24).
[41] Kositamongkol C, Kanchanasurakit S, Auttamalang C, et al. Coffee consumption and non-alcoholic fatty liver disease: an umbrella review and a systematic review and meta-analysis. Front Pharmacol 2021;12: 786596.
[42] Kennedy OJ, Fallowfield JA, Poole R, et al. All coffee types decrease the risk of adverse clinical outcomes in chronic liver disease: a UK Biobank study. BMC Public Health 2021;21(1):970.
[43] Pan A, Sun Q, Bernstein AM, et al. Red meat consumption and risk of type 2 diabetes: 3 cohorts of US adults and an updated meta-analysis. Am J Clin Nutr 2011;94(4):1088–1096.
[44] Zelber-Sagi S, Ivancovsky-Wajcman D, Fliss Isakov N, et al. High red and processed meat consumption is associated with non-alcoholic fatty liver disease and insulin resistance. J Hepatol 2018;68(6):1239–1246.
[45] Innes H, Morling JR, Buch S, et al. Performance of routine risk scores for predicting cirrhosis-related morbidity in the community. J Hepatol 2022;77(2):365–376.

Keywords: chronic liver disease; metabolic dysfunction-associated steatotic liver disease; cirrhosis; lifestyle; diet; polygenic risk.
Received 20 November 2023; received in revised form 4 April 2024; accepted 23 April 2024; Available online 26 April 2024

Journal of Hepatology, Volume *6*

Supplemental information

Lifestyle and genetic risk of chronic liver disease in metabolically healthy and unhealthy individuals from the general population

Isabel Drake, Alice Giontella, Mariam Miari, Kristina Önnerhag, and Marju Orho-Melander

Lifestyle and genetic risk of chronic liver disease in metabolically healthy and unhealthy individuals from the general population

Isabel Drake, Alice Giontella, Mariam Miari, Kristina Önnerhag, Marju Orho-Melander

Table of contents

Supplementary Data Description………………………………………………………….…2

Supplementary Tables…………………………………………………………………...5

Supplementary References………………………………………………………………17

Supplementary Data Description

Anthropometric, cardio-metabolic and blood measurements

At MDCS baseline examinations trained nurses measured height (m) and weight (kg) and body mass index (BMI) was calculated as weight divided by height squared (kg/m^2). Waist circumference (cm) was measured midway between the lowest rib margin and iliac crest. Blood pressure (mmHg) was measured using a mercury-column phygomanometer after 5 min of supine rest. Hypertension was defined as blood pressure >130/85 mmHg and/or use of anti-hypertensive medication(s). Prevalent diabetes mellitus at baseline was based on self-reported history of diabetes, diabetes diagnosis in national/local registries, current use of diabetes medications or fasting whole blood glucose of at least 6.1 mmol/l (corresponding to plasma glucose ≥7.0 mmol/L) at baseline examination.

All fasting blood samples were donated after an overnight fast and stored at −80°C. Fasting glucose, fasting insulin, high-density lipoprotein (HDL, mmol/l), and triglycerides (mmol/l) were measured at the Department of Clinical Chemistry, Skåne University Hospital in Malmö, which is attached to a national standardization system. Low-density lipoprotein (LDL) was estimated using Friedewald's formula. Fasting glucose at baseline was measured in whole blood by a hexokinase-glucose-6-phosphate dehydrogenase method (1). A constant factor of 1·11 was used to convert concentration in whole blood to the equivalent concentration in plasma (2). Homeostatic Model Assessment – Insulin Resistance (HOMA-IR) was calculated according to Matthews et al. (3) by using the formula: (fasting insulin × fasting glucose)/22.5, where insulin is expressed as mIU/l and glucose as mmol/l (1). C-reactive protein (CRP) concentration using the high-sensitive C-reactive protein (hsCRP) test, was performed using the Tina-quant® CRP latex assay (Roche Diagnostics, Basel, Switzerland) on an ADVIA® 1650 Chemistry System (Bayer healthcare, NY, USA).

Lifestyle variables

Age and sex were extracted from the participants' Swedish personal identification number. Educational level was categorized based on years and level of education completed i.e., less than 9 years or completed elementary school, middle school, high school or at least one year of studies at advanced level after high school but without degree, or university degree.

Smoking status was categorized as never, former or current (including irregular) based on self-reported use in the baseline questionnaire. Alcohol consumption was estimated based on information from both the baseline questionnaire and the reported intake during a 7-day food record that included detailed registration of cooked meals, medications, supplements and cold beverages (4). Non-consumers of alcohol (i.e., defined as those reporting no alcohol intake during the preceding year in the baseline questionnaire and reporting no intake during the 7-day registration) were classified as zero consumers while alcohol intake among consumers was categorized as low, moderate or high (i.e. <15, 15–30, or >30 g/day for women, and <20, 20–40, or >40 g/day for men). Level of leisure-time physical activity was assessed by participants reporting reported the number of minutes per week for seventeen different leisure-time activities and combined into a physical activity score (5). Participants were ranked from low to high leisure-time physical activity level by dividing them into sex-specific quartiles of total score.

Dietary intakes were assessed using a modified diet history method combining the 7-day food record with a 196-item semi-quantitative food questionnaire. Overall eating habits, quality of reported intakes in the food record and the questionnaire and potential overlap using the two modalities were further assessed using a 45-60 minutes dietary interview with a trained nutritionist (4). Reported food intake was used to calculate total dietary fiber intake using the food and nutrient data base from the Swedish National Food Agency (4). The reproducibility and validity of the diet assessment method has been described previously (6-8). We examined three previously proposed ‘healthy’ dietary components (dietary fiber, fruits and vegetables, and coffee) and two ‘unhealthy’ components (sugar-sweetened beverages and red and processed meat) dietary components. Dietary components examined were selected based on the previously reported directionally consistent associations with both liver-related outcomes and cardiometabolic diseases (9-12). Selection was further guided based on availability of data in the MDCS. Dietary intakes were energy-adjusted by calculating the relative intake in grams per 1000 kcal of estimated total energy intake.

Supplementary Tables

Table S1 ICD-codes and number of prevalent and incident first events of chronic liver disease (CLD) in the Malmö Diet and Cancer Study (N=30,446) identified in Swedish national registries including the inpatient register, hospital-based outpatient care and cause-of-death register. Only the first recorded event of the included endpoints is shown. Subjects with an incident diagnosis of chronic viral hepatitis and/or other specified cause of liver disease (n=82) were not included (ICD-10 B18, B19, E83-0, E83.1, K71, K74.3, K74.5, K75.2, K75.3, K75.4, K75.8, K75.9).

Diagnosis	ICD-10 codes	*n* incident	ICD-9 codes	*n* incident	*n* prevalent	Total *n* incident (ICD-9 + 10 codes)
Acute and subacute liver failure	K72.0	52	570	6	4	58
Chronic liver failure	K72.1	2	572.8	1	0	3
Liver failure	K72.9, K70.4,	116	-	-	-	116
Cirrhosis	K74.6, K70.3,	112	571.5	7	3	119
Portal hypertension	K76.6	6	572.3	2	0	8
Hepatorenal syndrome	K76.7	3	572.4	2	0	5
Esophageal varices	I85.0, I85.9	38	456.0, 456.20, 456.1, 456.21	11	10	49
Ascites*	R18.9, TJA10	32	789.5	0	3	32
Liver encephalopathy	-	-	572.2	0	0	1
Hepatocellular carcinoma	C22.0	48	155	12	2	75
Liver transplantation	JJC00, JJC10, JJC20, DJ005, DJ006, JJC30, JJC40	0	5051, 5059	0	0	0
**Total CLD**		410		41	22	451***

* Only ascites cases with a subsequent additional diagnosis of CLD were included.
** Of the total number of incident events, 82 cases (18.2%) were identified in the cause-of-death registry.

Table S2 List of genetic variants and weights used to construct the weighted polygenic risk scores (PRS) for MASLD (PRS-MASLD), cALT (PRS-cALT), and liver cirrhosis (PRS-cirrhosis).

PRS-MASLD				
Variant	Gene	Minor allele	MAF	Weight[a]
rs738408	*PNPLA3*	T	0.2098	32.721
rs8107974	*TM6SF2*	T	0.1033	22.859
rs2642442	*MARC1*	C	0.2987	-7.930
rs7029757	*TOR1B*	A	0.0902	-6,409
rs429358	*APOE*	C	0.1640	-12,374
rs10787429	*GPAM*	T	0.2838	8.656
rs140201358	*PNPLA2*	G	0.0174	5.480
rs62033400	*FTO*	G	0.4104	5.493
rs9303144	*SREBF1*	C	0.3001	5.675
rs626283	*TMC4/MBOAT7*	C	0.4256	6.15
rs8113542	*INSR*	G	0.2494	5.531
rs79953491	*COBLL1*	G	0.1245	-5.894
rs4665972	*GCKR*	T	0.3713	10.681
rs1229984	*ADH1B*	T	0.0215	-6.538
rs4423880*	*MTTP*	A	0.2576	-6.615
rs112875651	*TRIB1*	A	0.4002	-9.344
PRS-cirrhosis				
Variant	Gene	Minor allele	MAF	Weight[b]
rs738409	*PNPLA3*	G	0.2098	0.4886
rs58542926	*TM6SF2*	T	0.1028	0.3646
rs2642438	*MARC1*	A	0.2749	-0.0943
rs7029757	*TOR1B*	A	0.0902	-0.1625
rs429358	*APOE*	C	0.1640	-0.1625
rs28929474	*SERPINA1*	T	0.0286	0.7080
rs6834314	*HSD17B13*	G	0.3045	-0.1625
rs12904	*EFNA1*	A	0.4294	-0.1054
rs888655	*ARHGEF28*	A	0.2793	-0.0726
rs9398804	*CENPW*	A	0.4312	-0.0726
rs1006195**	*HMBS*	T	0.3829	0.2070
rs1883711	*MAFB*	C	0.0662	0.1906
PRS-cALT				
Variant	*Gene*	Minor allele	MAF	Weight[c]
rs738408	*PNPLA3*	T	0.2098	0.269
rs2642438	*MTARC1*	A	0.2752	-0.079
rs6734238	*IL1RN*	G	0.395	-0.059
rs13389219	*COBLL1; SCN2A*	T	0.424	-0.050
rs17036160	*PPARG*	T	0.1423	-0.073
rs10433937	*HSD17B13*	G	0.3039	-0.084
rs17598226	*MTTP*	G	0.2583	-0.041
rs4841132	*PPP1R3B*	A	0.0947	0.130
rs2980888	*lnc-TRIB1; WASHC5*	T	0.2696	0.139
rs10883451	*ERLIN1*	C	0.4695	-0.161
rs4918722	*GPAM*	C	0.285	0.075
rs28929474	*SERPINA1*	T	0.02876	0.481
rs56094641	*FTO*	G	0.4205	0.040
rs1801689	*APOH*	C	0.02567	0.176
rs11668950	*IFI30;MPV17L2;PIK3R*	A	0.2247	0.041
rs58542926	*TM6SF2*	T	0.1028	0.222

rs5117	*APOE;APOC1*	C	0.2422	-0.080

[a] Weights are the Z-scores from the GOLDPlus European ancestry meta-analysis presented in Chen et al. Nature Genetics 2023 (DOI: 10.1038/s41588-023-01497-6) (13). Negative weights were used if the reported risk-increasing allele was different from the minor allele.
[b] Weights are the natural log of odds ratios for liver cirrhosis from Emdin et al. Gastroenterology 2021 (DOI: 10.1053/j.gastro.2020.12.011) (14). Negative weights were used if the reported risk-increasing allele was different from the minor allele.
[c] Weights are the beta coefficients for unexplained chronically elevated ALT levels as a proxy for MASLD in Vojkuvic et al. Nature Genetics 2022 (DOI: 10.1038/s41588-022-01078-z) (15). Negative weights were used if the reported risk-increasing allele was different from the minor allele.
* Proxy variant for rs138764179 identified by Chen et al. (13)
** Proxy variant for rs1799992 identified by Emdin et al. (14)

Table S3 Cardiometabolic risk factors for chronic liver disease (CLD) in the Malmö Diet and Cancer Study (MDCS; *n*=27,991) and the sub-sample with fasting blood samples taken at baseline (*n*=4,549).

Risk factor	Model*	HR	95% CI	*p* value
Prevalent diabetes mellitus (yes/no)	Model 1	2.53	1.79-3.57	1.4×10^{-7}
	Model 2	2.22	1.56-3.15	8.3×10^{-6}
Hypertension (yes/no)	Model 1	1.30	1.05-1.61	1.4×10^{-2}
	Model 2	1.15	0.92-1.43	0.21
Lipid-lowering drugs (yes/no)	Model 1	1.23	0.74-2.04	0.42
	Model 2	1.10	0.66-1.82	0.72
Body mass index, per SD increase	Model 1	1.32	1.19-1.46	2.3×10^{-7}
	Model 2	1.26	1.13-1.40	2.6×10^{-5}
Waist circumference, per SD increase	Model 1	1.59	1.40-1.81	4.6×10^{-13}
	Model 2	1.93	1.49-2.50	6.3×10^{-7}
		Sub-sample only		
Fasting glucose, per SD increase	Model 1	1.64	0.88-3.08	0.12
	Model 2	1.43	0.74-2.74	0.29
HbA1c, per SD increase	Model 1	1.14	0.72-1.80	0.58
	Model 2	1.10	0.69-1.74	0.69
HOMA-IR, per SD increase	Model 1	2.02	1.58-2.58	1.8×10^{-8}
	Model 2	2.11	1.62-2.75	2.8×10^{-8}
LDL, per SD increase	Model 1	0.86	0.63-1.16	0.31
	Model 2	0.83	0.61-1.13	0.23
HDL, per SD increase	Model 1	0.84	0.60-1.18	0.32
	Model 2	0.91	0.64-1.30	0.61
Triglycerides, per SD increase	Model 1	1.34	0.98-1.81	0.063
	Model 2	1.26	0.91-1.73	0.17
hsCRP, per SD increase	Model 1	1.35	1.01-1.82	0.046
	Model 2	1.28	0.94-1.75	0.12

* Model 1 presents hazard ratios (HR) and 95% confidence intervals from a Cox proportional hazards regression model adjusting for age and sex. Model 2 includes adjustment for age, sex, prevalent diabetes mellitus, body mass index, hypertension and use of lipid-lowering drugs.

Table S4 Multiplicative interaction terms between cardiometabolic, lifestyle and genetic risk factors on risk of chronic liver disease from a Cox proportional hazards regression model with adjustment for age, sex, and educational level. Nominally significant interaction terms ($p<0.05$) are marked in bold font.

Risk factors	*PNPLA3* rs738409	PRS-MASLD	PRS-cirrhosis	PRS-cALT
**Metabolic health status**	**0.85 (0.75-0.97)***	1.00 (0.92-1.08)	0.97 (0.90-1.04)	1.00 (0.92-1.08)
Prevalent diabetes mellitus	**0.54 (0.30-0.99)***	1.01 (0.73-1.40)	1.06 (0.77-1.45)	1.25 (0.91-1.70)
Body mass index	**0.81 (0.69-0.96)***	0.99 (0.89-1.10)	1.00 (0.91-1.09)	1.02 (0.93-1.13)
Waist circumference	0.89 (0.76-1.05)	1.05 (0.94-1.16)	1.05 (0.95-1.16)	1.05 (0.95-1.16)
Hypertension	0.79 (0.57-1.08)	0.98 (0.79-1.20)	0.90 (0.74-1.09)	0.96 (0.79-1.17)
Use of lipid-lowering drugs	0.84 (0.37-1.90)	0.86 (0.52-1.44)	0.83 (0.50-1.37)	0.84 (0.51-1.39)
**Lifestyle risk score**	1.06 (0.82-1.39)	1.07 (0.90-1.26)	0.94 (0.80-1.10)	0.97 (0.82-1.14)
Smoking status	1.15 (0.95-1.39)	1.09 (0.97-1.23)	1.01 (0.90-1.13)	0.99 (0.89-1.11)
Alcohol consumption	1.09 (0.95-1.26)	1.02 (0.93-1.12)	1.02 (0.93-1.11)	1.01 (0.93-1.11)
Physical activity	0.91 (0.79-1.04)	1.00 (0.91-1.09)	1.00 (0.92-1.09)	0.95 (0.87-1.03)
Diet risk score	1.22 (0.92-1.62)	**1.25 (1.04-1.49)***	1.05 (0.88-1.24)	1.14 (0.96-1.36)
Dietary fiber	**0.83 (0.71-0.97)***	0.91 (0.82-1.00)	0.93 (0.85-1.03)	1.01 (0.92-1.11)
Fruit and vegetables	0.92 (0.81-1.06)	0.98 (0.89-1.08)	1.00 (0.92-1.09)	1.05 (0.96-1.14)
SSB	1.00 (0.86-1.17)	**1.12 (1.02-1.24)***	1.08 (0.98-1.19)	1.08 (0.98-1.18)
Coffee	0.98 (0.85-1.14)	0.98 (0.89-1.07)	1.00 (0.92-1.09)	0.96 (0.88-1.05)
Red/processed meat	0.95 (0.80-1.13)	1.03 (0.92-1.15)	0.97 (0.87-1.08)	1.01 (0.90-1.13)

* $p<0.05$

Table S5 Effect of genetic risk variants (single nucleotide polymorphism; SNP) included in polygenic risk scores on risk of chronic liver disease (CLD) in the MDCS (N=26,965). Hazard ratios (HR) and 95% confidence intervals (CI) from a Cox proportional hazards regression model adjusting for age and sex.

Gene	SNP	PRS	Genotype	HR (95% CI)	*p* value
PNPLA3	rs738409	Cirrhosis	CC	1.00 (ref)	
			CG	1.20 (0.97-1.50)	0.099
			GG	2.31 (1.61-3.32)	6.3×10^{-6}
			Per allele effect	1.38 (1.17-1.63)	1.0×10^{-4}
PNPLA3	rs738408	MASLD, cALT	CC	1.00 (ref)	
			CT	1.20 (0.97-1.50)	0.099
			TT	2.31 (1.61-3.32)	6.3×10^{-6}
			Per allele effect	1.38 (1.17-1.63)	1.0×10^{-4}
TM6SF2	rs58542926	Cirrhosis, cALT	CC	1.00 (ref)	
			CT	1.16 (0.90-1.49)	0.26
			TT	3.19 (1.75-5.83)	1.6×10^{-4}
			Per allele effect	1.34 (1.08-1.66)	8.3×10^{-3}
TM6SF2	rs8107974	MASLD	AA	1.00 (ref)	
			AT	1.15 (0.90-1.49)	0.27
			TT	3.09 (1.69-5.64)	2.4×10^{-4}
			Per allele effect	1.33 (1.07-1.64)	9.7×10^{-3}
GCKR	rs4665972	MASLD	CC	1.00 (ref)	
			CT	0.93 (0.75-1.15)	0.5
			TT	0.82 (0.59-1.16)	0.26
			Per allele effect	0.91 (0.78-1.06)	0.25
TMC4/MBOAT7	rs626283	MASLD	GG	1.00 (ref)	
			GC	1.02 (0.81-1.29)	0.84
			CC	1.15 (0.86-1.54)	0.34
			Per allele effect	1.07 (0.92-1.24)	0.38
SERPINA1	rs28929474	Cirrhosis, cALT	CC	1.00 (ref)	
			CT	1.71 (1.20-2.43)	2.9×10^{-3}
			TT	9.51 (2.37-38.20)	1.5×10^{-3}
			Per allele effect	1.85 (1.33-2.56)	2.6×10^{-4}
HSD17B13	rs6834314	Cirrhosis	AA	1.00 (ref)	
			AG	0.85 (0.68-1.05)	0.13
			GG	0.75 (0.51-1.11)	0.15
			Per allele effect	0.86 (0.73-1.01)	0.065
HSD17B13	rs10433937	cALT	TT	1.00 (ref)	
			TG	0.84 (0.68-1.05)	0.13

			GG	0.73 (0.49-1.08)	0.12
			Per allele effect	0.85 (0.72-1.00)	0.049
MARC_1	rs2642438	Cirrhosis, cALT	GG	1.00 (ref)	
			GA	0.97 (0.78-1.20)	0.79
			AA	0.78 (0.51-1.21)	0.27
			Per allele effect	0.93 (0.79-1.09)	0.36
MARC_1	rs2642442	MASLD	TT	1.00 (ref)	
			TC	0.92 (0.74-1.14)	0.47
			CC	0.83 (0.56-1.22)	0.34
			Per allele effect	0.92 (0.78-1.07)	0.28
EFNA1	rs12904	Cirrhosis	GG	1.00 (ref)	
			GA	0.86 (0.69-1.08)	0.2
			AA	0.72 (0.52-0.98)	0.036
			Per allele effect	0.85 (0.73-0.99)	0.031
ARHGEF28	rs888655	Cirrhosis	GG	1.00 (ref)	
			AG	0.92 (0.74-1.13)	0.43
			AA	0.51 (0.30-0.84)	8.7×10^{-3}
			Per allele effect	0.82 (0.69-0.97)	0.021
CENPW	rs9398804	Cirrhosis	TT	1.00 (ref)	
			TA	1.17 (0.92-1.48)	0.21
			AA	1.12 (0.82-1.51)	0.48
			Per allele effect	1.07 (0.92-1.24)	0.38
TOR1B	rs7029757	MASLD	GG	1.00 (ref)	
			GA	1.04 (0.79-1.36)	0.8
			AA	0.38 (0.05-2.73)	0.34
			Per allele effect	0.98 (0.76-1.27)	0.89
HMBS	rs1006195	Cirrhosis	GG	1.00 (ref)	
			GT	1.17 (0.93-1.47)	0.18
			TT	1.35 (0.99-1.82)	0.055
			Per allele effect	1.16 (1.00-1.35)	0.046
APOE	rs429358	MASLD, Cirrhosis	TT	1.00 (ref)	
			TC	0.88 (0.69-1.12)	0.28
			CC	1.03 (0.55-1.94)	0.92
			Per allele effect	0.92 (0.75-1.13)	0.42
APOE; APOC1	rs5117	cALT	TT	1.00 (ref)	
			TC	1.00 (0.81-1.25)	0.91
			CC	0.73 (0.44-1.22)	0.23
			Per allele effect	0.94 (0.79-1.11)	0.46
APOH	rs1801689	cALT	AA	1.00 (ref)	

			AC	1.32 (0.86-2.01)	0.2
			CC	8.41 (2.09-33.80)	2.7×10^{-3}
			Per allele effect	1.49 (1.02-2.19)	0.041
MAFB	rs1883711	Cirrhosis	GG	1.00 (ref)	
			GC	0.78 (0.56-1.10)	0.16
			CC	0.58 (0.08-4.13)	0.59
			Per allele effect	0.78 (0.57-1.08)	0.13
GPAM	rs10787429	MASLD	CC	1.00 (ref)	
			CT	1.13 (0.91-1.40)	0.26
			TT	1.04 (0.70-1.53)	0.86
			Per allele effect	1.06 (0.91-1.24)	0.45
GPAM	rs4918722	cALT	TT	1.00 (ref)	
			TC	1.15 (0.92-1.42)	0.21
			CC	1.00 (0.67-1.48)	0.98
			Per allele effect	1.06 (0.90-1.24)	0.49
PNPLA2	rs140201358	MASLD	CC	1.00 (ref)	
			CG/GG*	0.85 (0.47-1.55)	0.6
			Per allele effect	0.85 (0.47-1.54)	0.59
FTO	rs62033400	MASLD	AA	1.00 (ref)	
			AG	1.08 (0.86-1.36)	0.5
			GG	1.08 (0.80-1.47)	0.61
			Per allele effect	1.05 (0.90-1.21)	0.54
FTO	rs56094641	cALT	AA	1.00 (ref)	
			AG	1.08 (0.86-1.37)	0.5
			GG	1.08 (0.80-1.46)	0.62
			Per allele effect	1.05 (0.90-1.21)	0.55
SREBF1	rs9303144	MASLD	TT	1.00 (ref)	
			TC	0.99 (0.80-1.23)	0.96
			CC	1.04 (0.72-1.51)	0.84
			Per allele effect	1.01 (0.86-1.18)	0.91
INSR	rs8113542	MASLD	AA	1.00 (ref)	
			AG	1.06 (0.86-1.32)	0.58
			GG	0.92 (0.58-1.45)	0.71
			Per allele effect	1.01 (0.86-1.20)	0.89
COBLL1	rs79953491	MASLD	AA	1.00 (ref)	
			AG	1.12 (0.88-1.43)	0.35
			GG	0.53 (0.17-1.66)	0.28
			Per allele effect	1.03 (0.83-1.28)	0.78
COBLL1; SCN2A	rs13389219	cALT	CC	1.00 (ref)	

			CT	0.94 (0.75-1.17)	0.56
			TT	0.81 (0.59-1.10)	0.18
			Per allele effect	0.91 (0.78-1.05)	0.19
ADH1B	rs1229984*	MASLD	CC	1.00 (ref)	
			CT/TT	0.63 (0.33-1.23)	0.17
			Per allele effect	0.63 (0.33-1.19)	0.16
MTTP	rs4423880	MASLD	GG	1.00 (ref)	
			GA	1.09 (0.88-1.35)	0.45
			AA	1.08 (0.71-1.63)	0.73
			Per allele effect	1.06 (0.90-1.25)	0.48
MTTP	rs17598226	cALT	CC	1.00 (ref)	
			CG	1.05 (0.85-1.31)	0.64
			GG	1.06 (0.70-1.61)	0.78
			Per allele effect	1.04 (0.88-1.23)	0.63
TRIB1	rs112875651	MASLD	GG	1.00 (ref)	
			GA	1.11 (0.89-1.40)	0.35
			AA	0.97 (0.71-1.34)	0.87
			Per allele effect	1.01 (0.87-1.17)	0.88
IL1RN	rs6734238	cALT	AA	1.00 (ref)	
			AG	1.01 (0.80-1.27)	0.95
			GG	1.18 (0.87-1.59)	0.28
			Per allele effect	1.07 (0.92-1.24)	0.36
PPARG	rs17036160	cALT	CC	1.00 (ref)	
			CT	0.93 (0.73-1.19)	0.58
			TT	0.64 (0.26-1.55)	0.33
			Per allele effect	0.90 (0.72-1.12)	0.33
PPP1R3B	rs4841132	cALT	GG	1.00 (ref)	
			GA	1.02 (0.78-1.34)	0.86
			AA	1.15 (0.43-3.08)	0.78
			Per allele effect	1.03 (0.81-1.32)	0.79
lnc-TRIB1; WASHC5	rs2980888	cALT	CC	1.00 (ref)	
			CT	1.22 (0.99-1.51)	0.061
			TT	0.60 (0.35-1.02)	0.058
			Per allele effect	1.00 (0.85-1.17)	0.96
ERLIN1	rs10883451	cALT	TT	1.00 (ref)	
			TC	0.89 (0.70-1.13)	0.32
			CC	0.90 (0.68-1.20)	0.48
			Per allele effect	0.94 (0.82-1.09)	0.44

IFI30;MPV17L2;PIK3R	rs11668950	cALT	GG	1.00 (ref)	
			GA	1.10 (0.89-1.37)	0.38
			AA	1.00 (0.62-1.61)	0.99
			Per allele effect	1.05 (0.89-1.25)	0.55

* Homozygous carriers of the minor allele were few and therefore heterozygous and homozygous carriers of the minor allele were collapsed into one category.

Table S6 *PNPLA3* rs738409 genetic risk variant and polygenic risk scores (PRSs) for metabolic dysfunction-associated steatotic liver disease (MASLD), liver cirrhosis and unexplained chronic ALT elevation (cALT) in relation to incidence of chronic liver disease (CLD), steatotic liver disease (unspecified), liver cirrhosis (all-cause) and hepatocellular carcinoma (HCC) in the Malmö Diet and Cancer Study (N=26,965) stratified by age and sex. Hazard ratios (HR) and 95% confidence intervals (CI) per risk G-allele in *PNPLA3* rs738409 and per standard deviation increase in normalized (z-score) PRS-MASLD, PRS-cirrhosis and PRS-cALT.

	CLD (n_{cases}=365)		Steatotic liver disease (n_{cases}=76)		Liver cirrhosis (n_{cases}=173)		HCC (n_{cases}=72)	
	HR (95% CI)	*p* value	HR (95% CI)	*p* value	HR (95% CI)	*p* value	HR (95% CI)	*p* value
PNPLA3 rs738409								
All	1.38 (1.17-1.62)	1.2×10^{-4}	1.39 (0.97-1.98)	0.073	1.79 (1.43-2.24)	3.5×10^{-7}	1.93 (1.37-2.72)	1.7×10^{-4}
Men	1.34 (1.08-1.66)	7.3×10^{-3}	1.57 (0.88-2.80)	0.13	1.65 (1.24-2.20)	5.8×10^{-4}	1.86 (1.22-2.83)	3.9×10^{-3}
Women	1.43 (1.11-1.84)	6.0×10^{-3}	1.29 (0.82-2.03)	0.27	2.03 (1.41-2.91)	1.2×10^{-4}	2.06 (1.14-3.71)	0.016
Age <60 years	1.45 (1.17-1.79)	7.4×10^{-4}	1.72 (1.17-2.53)	0.0061	2.05 (1.56-2.70)	2.5×10^{-7}	1.95 (1.20-3.16)	6.6×10^{-3}
Age ≥60 years	1.31 (1.01-1.68)	0.038	0.49 (0.17-1.37)	0.17	1.38 (0.93-2.05)	0.11	1.94 (1.19-3.17)	7.9×10^{-3}
PRS-MASLD								
All	1.23 (1.11-1.35)	5.6×10^{-5}	1.25 (1.01-1.55)	0.043	1.45 (1.26-1.67)	1.5×10^{-7}	1.52 (1.24-1.90=	1.0×10^{-4}
Men	1.24 (1.10-1.41)	8.0×10^{-4}	1.23 (0.86-1.77)	0.26	1.40 (1.17-1.67)	1.9×10^{-4}	1.66 (1.28-2.15)	1.2×10^{-4}
Women	1.20 (1.03-1.40)	2.1×10^{-2}	1.26 (0.96-1.65)	0.094	1.55 (1.24-1.95)	1.6×10^{-4}	1.28 (0.87-1.89)	0.20
Age <60 years	1.29 (1.13-1.47)	1.5×10^{-4}	1.48 (1.16-1.88)	0.0013	1.62 (1.36-1.92)	4.3×10^{-8}	1.51 (1.11-2.04)	8.3×10^{-3}
Age ≥60 years	1.15 (0.99-1.34)	0.064	0.65 (0.39-1.09)	0.10	1.20 (0.94-1.52)	0.14	1.56 (1.15-2.12)	3.8×10^{-3}
PRS-cirrhosis								
All	1.36 (1.24-1.50)	8.9×10^{-11}	1.38 (1.13-1.70)	1.8×10^{-3}	1.65 (1.45-1.87)	1.5×10^{-14}	1.80 (1.49-2.18)	1.9×10^{-9}
Men	1.42 (1.26-1.60)	9.3×10^{-9}	1.14 (0.79-1.64)	0.47	1.64 (1.39-1.92)	1.5×10^{-9}	2.06 (1.65-2.56)	1.0×10^{-10}
Women	1.28 (1.10-1.49)	1.2×10^{-3}	1.53 (1.19-1.95)	7.8×10^{-4}	1.66 (1.35-2.05)	2.0×10^{-6}	1.28 (0.88-1.86)	0.20
Age <60 years	1.38 (1.22-1.56)	3.8×10^{-7}	1.53 (1.22-1.91)	2.4×10^{-4}	1.72 (1.47-2.01)	1.1×10^{-11}	1.97 (1.52-2.54)	2.7×10^{-7}
Age ≥60 years	1.34 (1.16-1.55)	4.8×10^{-5}	0.96 (0.60-1.54)	0.88	1.53 (1.23-1.90)	1.4×10^{-4}	1.63 (1.23-2.17)	7.2×10^{-4}
PRS-cALT								
All	1.34 (1.21-1.47)	2.9×10^{-9}	1.11 (0.89-1.38)	0.35	1.54 (1.35-1.76)	2.1×10^{-10}	1.59 (1.29-1.95)	9.8×10^{-6}

Men	1.38 (1.22-1.56)	3.3×10^{-7}	0.98 (0.67-1.43)	0.91	1.59 (1.35-1.88)	5.0×10^{-8}	1.77 (1.39-2.26)	4.6×10^{-6}
Women	1.26 (1.08-1.47)	2.5×10^{-3}	1.19 (0.91-1.55)	0.21	1.44 (1.15-1.80)	1.4×10^{-3}	1.23 (0.84-1.80)	0.28
Age <60 years	1.36 (1.20-1.55)	2.3×10^{-6}	1.13 (0.88-1.45)	0.34	1.56 (1.32-1.84)	2.1×10^{-7}	1.80 (1.36-2.38)	3.7×10^{-5}
Age ≥60 years	1.30 (1.13-1.50)	2.9×10^{-4}	1.06 (0.67-1.65)	0.81	1.52 (1.22-1.89)	2.2×10^{-4}	1.39 (1.03-1.87)	0.031

Supplementary References

1. Hedblad B, Nilsson P, Janzon L, Berglund G. Relation between insulin resistance and carotid intima-media thickness and stenosis in non-diabetic subjects. Results from a cross-sectional study in Malmo, Sweden. Diabet Med. 2000;17(4):299-307.
2. D'Orazio P, Burnett RW, Fogh-Andersen N, Jacobs E, Kuwa K, Kulpmann WR, et al. Approved IFCC recommendation on reporting results for blood glucose: International Federation of Clinical Chemistry and Laboratory Medicine Scientific Division, Working Group on Selective Electrodes and Point-of-Care Testing (IFCC-SD-WG-SEPOCT). Clin Chem Lab Med. 2006;44(12):1486-90.
3. Matthews DR, Hosker JP, Rudenski AS, Naylor BA, Treacher DF, Turner RC. Homeostasis model assessment: insulin resistance and beta-cell function from fasting plasma glucose and insulin concentrations in man. Diabetologia. 1985;28(7):412-9.
4. Wirfalt E, Mattisson I, Johansson U, Gullberg B, Wallstrom P, Berglund G. A methodological report from the Malmo Diet and Cancer study: development and evaluation of altered routines in dietary data processing. Nutr J. 2002;1:3.
5. Mattisson I, Wirfalt E, Aronsson CA, Wallstrom P, Sonestedt E, Gullberg B, et al. Misreporting of energy: prevalence, characteristics of misreporters and influence on observed risk estimates in the Malmo Diet and Cancer cohort. Br J Nutr. 2005;94(5):832-42.
6. Elmstahl S, Gullberg B, Riboli E, Saracci R, Lindgarde F. The Malmo Food Study: the reproducibility of a novel diet history method and an extensive food frequency questionnaire. Eur J Clin Nutr. 1996;50(3):134-42.
7. Elmstahl S, Riboli E, Lindgarde F, Gullberg B, Saracci R. The Malmo Food Study: the relative validity of a modified diet history method and an extensive food frequency questionnaire for measuring food intake. Eur J Clin Nutr. 1996;50(3):143-51.
8. Riboli E, Elmstahl S, Saracci R, Gullberg B, Lindgarde F. The Malmo Food Study: validity of two dietary assessment methods for measuring nutrient intake. Int J Epidemiol. 1997;26 Suppl 1:S161-73.

9. Hassani Zadeh S, Mansoori A, Hosseinzadeh M. Relationship between dietary patterns and non-alcoholic fatty liver disease: A systematic review and meta-analysis. J Gastroenterol Hepatol 2021; 36(6):1470-1478.

10. Kennedy OJ, Fallowfield JA, Poole R, Hayes PC, Parkes J, Roderick PJ. All coffee types decrease the risk of adverse clinical outcomes in chronic liver disease: a UK Biobank study. BMC Public Health. 2021; 21(1): 970.

11. Carlström M, Larsson SC. Coffee consumption and reduced risk of developing type 2 diabetes: a systematic review with meta-analysis. Nutr Rev. 2018; 76(6):395-417.

12. Miller V, Micha R, Choi E, Karageorgou D, Webb P, Mozaffarian D. Evaluation of the Quality of Evidence of the Association of Foods and Nutrients With Cardiovascular Disease and Diabetes: A Sytematic Review. JAMA Netw Open. 2022; 5(2):e2146705.

13. Chen Y, Du X, Kuppa A, Feitosa MF, Bielak LF, O'Connell JR, et al. Genome-wide association meta-analysis identifies 17 loci associated with nonalcoholic fatty liver disease. Nat Genet. 2023; 55(10):1640-1650.

14. Emdin CA, Haas M, Ajmera V, Simon TG, Homburger J, Neben C, et al. Association of Genetic Variation With Cirrhosis: A Multi-Trait Genome-Wide Association and Gene-Environment Interaction Study. Gastroenterology. 2021;160(5):1620-33 e13.

15. Vujkovic M, Ramdas S, Lorenz KM, Guo X, Darlay R, Cordell HJ, et al. A multiancestry genome-wide association study of unexplained chronic ALT elevation as a proxy for nonalcoholic fatty liver disease with histological and radiological validation. Nat Genet. 2022; 54(6):761-771.
